# NEURONpyxl: fast, flexible, Python-integrated simulation of biophysical neural networks with complex plastic synapses

**DOI:** 10.3389/fncom.2026.1771884

**Published:** 2026-05-19

**Authors:** Uri Dickman, Peter J. Thomas, Hillel J. Chiel, John H. Byrne, Curtis L. Neveu

**Affiliations:** 1 Department of Mechanical Engineering, University of California, Santa Barbara, Santa Barbara, CA, United States; 2 Department of Mathematics, Applied Mathematics, and Statistics, Case Western Reserve University, Cleveland, OH, United States; 3 Department of Biology, Case Western Reserve University, Cleveland, OH, United States; 4 Department of Cognitive Science, Case Western Reserve University, Cleveland, OH, United States; 5 Department of Electrical, Computer, and Systems Engineering, Case Western Reserve University, Cleveland, OH, United States; 6 Department of Computer and Data Sciences, Case Western Reserve University, Cleveland, OH, United States; 7 Department of Neurosciences, Case Western Reserve University, Cleveland, OH, United States; 8 Department of Biomedical Engineering, Case Western Reserve University, Cleveland, OH, United States; 9 Department of Neurobiology and Anatomy, W.M. Keck Center for the Neurobiology of Learning and Memory, McGovern Medical School at the University of Texas Health Science Center, Houston, TX, United States

**Keywords:** *Aplysia californica*, central pattern generators, conductance-based modeling, NEURON, Python, SNNAP

## Abstract

**Introduction:**

NEURON has been widely used as an empirically-based simulation tool, especially for multi-compartment conductance-based neuronal modeling. The network mediating feeding in Aplysia californica has been extensively studied as a model central pattern generator. Understanding the relationship between network parameter values and their effect on animal behavior is of key importance in systems such as the Aplysia feeding apparatus, where detailed biophysical models can be constructed.

**Objective:**

This study aims to develop a new Python tool called NEURONpyxl that reads parameters from a spreadsheet to construct full neural networks to make it easier to create complex models in the NEURON simulation environment, incorporating short-term forms of plasticity such as depression or facilitation.

**Methods:**

Test simulations from well-understood networks were created in NEURONpyxl, and compared to simulation results of the same network in another neural simulator, the Simulator for Neural Networks and Action Potentials (SNNAP), which has previously been used to model conductance-based networks that include complex synaptic connections and multiple forms of synaptic plasticity. NEURONpyxl was then used to conduct a parameter grid search to optimize conductances in a previously developed network model of Aplysia feeding behavior.

**Results:**

Simulations of the test networks in NEURONpyxl and SNNAP produced numerically equivalent results, with differences remaining within the expected margin of error arising from numerical integration and implementation details. We then located parameter values that generated simulated motor patterns with durations of protraction and retraction that matched biological feeding behavior under different mechanical loads.

**Conclusion:**

NEURONpyxl simplifies building and simulating complex neural networks with different forms of synaptic plasticity, and locating physiologically relevant parameter values. With NEURONpyxl, future work may include the creation of ensembles of network models and the integration of biomechanics with complex conductance-based networks.

## 1 Introduction

Understanding the relationship between neural networks and the behaviors they mediate is an important area of investigation for computational neuroscience. Many behaviors are mediated by central pattern generators (CPGs) that control processes such as respiration (Butera et al., 1999), heartbeat rhythm (Lamb and Calabrese, 2011), and locomotion ranging from swimming in lampreys (Sigvardt and Williams, 1992) to walking in humans (Guertin, 2013). Extensive work has improved the understanding of CPG networks (Wang and Rinzel, 1992; Skinner et al., 1994; Manor et al., 2003; Baxter and Byrne, 2006). However, it is often challenging to relate CPG circuit parameters to quantitative metrics of natural animal behavior (Bässler, 1986; Bässler and Büschges, 1998; Diekman et al., 2017, 2024).

One model species used in neuroscience to study CPG networks is *Aplysia californica*. *Aplysia* feeding consists of a variety of well-defined behaviors (e.g., biting, swallowing, rejection) (Kupfermann, 1974) that are mediated by electrophysiologically accessible neurons, making this system ideal for neural analysis (Morton and Chiel, 1993b; Sutton et al., 2004; Cropper et al., 2004; Baxter and Byrne, 2006). These behaviors are due to the activation of key groups of motor neurons within the buccal ganglia that innervate key muscles of the feeding apparatus (buccal mass) (Morton and Chiel, 1993a,b; Church and Lloyd, 1994). Recordings in intact animals have identified these motor neurons and characterized their functional role in biting, swallowing, and rejection (Morton and Chiel, 1993a,b; Hurwitz et al., 1996; Ye et al., 2006a). The pattern generator is of general interest because it is multifunctional, flexible, and subject to learning, and thus is a good model system for illustrating the power of conductance-based models to understand the biophysical and cellular mechanisms of these properties.

The pattern of activity of neurons that mediate feeding behavior is called a buccal motor program (BMP), which consists of two distinct phases: protraction, mediated by the B31/32 multi-action neurons and other protraction motor neurons (Susswein and Byrne, 1988; Hurwitz et al., 1996; Church and Lloyd, 1994), and retraction, mediated by interneuron B64 and retraction motor neurons (Morton and Chiel, 1993b; Hurwitz and Susswein, 1996). During ingestion (i.e., bites and swallows), the grasper (muscular structure in the buccal mass that operates feeding *Aplysia*) is open during the protraction phase and closed during the retraction phase due to the activation of the B8a/b closure motor neurons (Morton and Chiel, 1993a,b). In contrast, during rejection, as the grasper is protracted, the B8a/b motor neurons close the grasper, pushing inedible material out of the buccal cavity, and the grasper is then retracted while open (Morton and Chiel, 1993b; Ye et al., 2006b). Even within a single type of ingestion behavior (i.e., swallowing), there can be a range of retraction responses with variations occurring spontaneously (Ye et al., 2006a), or in response to increased mechanical load (Gill and Chiel, 2020).

Conductance-based computational models for the neural circuit mediating *Aplysia* feeding (Costa et al., 2020; Momohara et al., 2022) have been previously constructed using the Simulator for Neural Networks and Action Potentials (SNNAP) (Ziv et al., 1994; Baxter and Byrne, 2007). SNNAP is a standalone Java program that integrates a set of ordinary differential equations whose parameters are defined in ASCII files. SNNAP includes a wide-range of capabilities including conductance-based modeling, ion pools, current and voltage clamping, and chemical neuromodulation due to the activation of second messenger systems. For example, SNNAP has been used to model the modulation of voltage-gated ion channels by the accumulation of second messengers such as Ca^2^+ and cAMP (Baxter et al., 1999; Cai et al., 2003; Burrell and Crisp, 2008; Neveu et al., 2023).

SNNAP is no longer under development and is difficult to integrate with modern machine learning tools. A MATLAB-based tool has previously been developed to automatically generate the SNNAP ASCII files from a Microsoft Excel spreadsheet (Momohara et al., 2022). Here, we present a new Python package that integrates NEURON, Python, and spreadsheet-based parameter organization, which we call NEURONpyxl.

NEURON is widely used and incorporates detailed biophysical modeling (Carnevale and Hines, 2006), and can be easily integrated with Python-based machine learning tools (Hussain et al., 2024; Orozco Valero et al., 2025). In addition, NEURON can be useful in constructing neuromechanical models (Fietkiewicz et al., 2023). Having a single simulation platform for neuromechanical modeling with detailed multi-conductance neural circuits would be a clear advantage as new neuromechanical models are developed (Lyttle et al., 2017; Webster-Wood et al., 2020; Yu and Thomas, 2021; Li et al., 2024; Sukhnandan et al., 2024). Alternative platforms for computational modeling of neural networks include NEST (Gewaltig and Diesmann, 2007), PyNN (Davison et al., 2009), GENESIS (Bower and Beeman, 2012), SONATA (Dai et al., 2020), Brian2 (Stimberg et al., 2019), and NetPyNE (Dura-Bernal et al., 2019).

In this work, we showed that NEURONpyxl and SNNAP models produce equivalent results for the same model. Next, we applied a parameter grid search to find parameters of a previously generated CPG model (Momohara et al., 2022) that produces BMPs that match the duration of protraction and retraction in ingestive BMPs in response to the presence or absence of mechanical load (Gill and Chiel, 2020). Thus, we demonstrate a method to easily read in and optimize parameters for a complex multi-conductance model using modern computational tools.

## 2 Methods

### 2.1 Computational approaches

SNNAP implements Hodgkin-Huxley type conductance-based modeling (Ziv et al., 1994; Baxter and Byrne, 2007). In SNNAP and NEURONpyxl, neurons are modeled without cell morphology (point cells); and, as with many NEURON models, without temperature dependence. The membrane current of each neuron compartment is comprised of current from voltage-dependent ion channels (*I*^(vd)^), electrical synapses (*I*^(es)^), chemical synapses (*I*^(cs)^), and exogenous current (*I*^app^). The total membrane current of the *i*^th^ cell in the network is given by:


$$\begin{array}{l} C_{\text{m}}\frac{dV_{i}}{dt}=I_{i}^{\text{(app)}}-\underset{j}{\sum }I_{ij}^{\text{(vd)}}-\underset{k}{\sum }I_{ik}^{\text{(es)}}-\underset{l}{\sum }I_{il}^{\text{(cs)}} \end{array}$$ (1)

**Figure 1:**
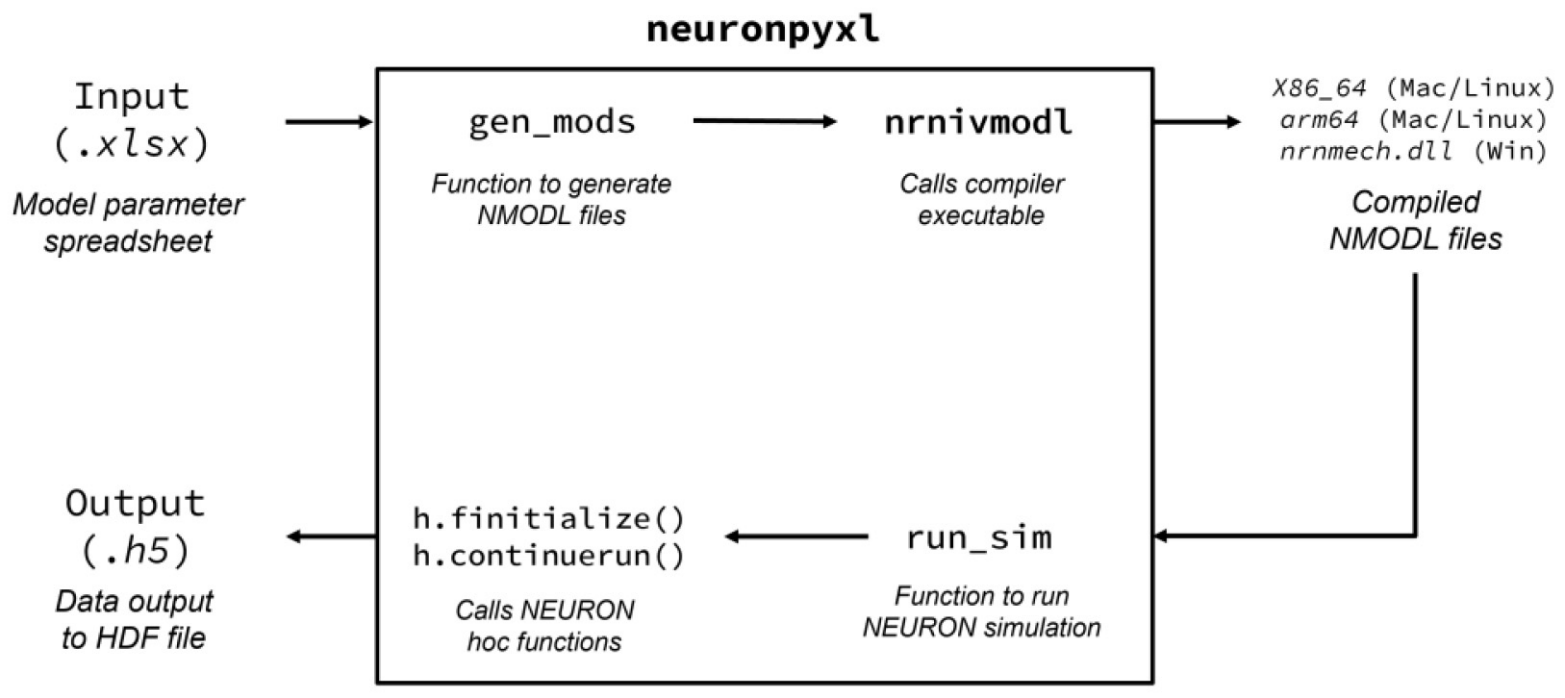
Workflow of the NEURONpyxl program run through the command-line interface. The two possible commands a user can run are neuronpyxl -f gen_mods –file path/to/excel/file.xlsx and neuronpyxl -f run_sim –file path/to/excel/file.xlsx –name simname –duration simdur args. Users interface with the command line through the Python API entrypoint via the neuronpyxl command, which generates and compiles MOD files from an Excel spreadsheet, or runs compiled files and save the output. (Alternatively, NEURONpyxl can be controlled directly through the Python API in order to further customize execution.) The gen_mods function generates the NMODL files required for custom NEURON simulations, and the run_sim function runs a single NEURON simulation by constructing a Network class object, and executing the functions within the Network object to run the NEURON solvers.

The term $\underset{j}{\sum }I_{ij}^{\text{(vd)}}$ is the sum of voltage-dependent currents from ion channel *j* into cell *i*, $\underset{k}{\sum }I_{ik}^{\text{(es)}}$ is the sum of currents into cell *i* due to electrical synapses from cell *k*, and $\underset{l}{\sum }I_{il}^{\text{(cs)}}$ is the sum of currents into cell *i* due to chemical synapses from cell *l*. The current due to a voltage-dependent conductance ($I_{ij}^{\text{(vd)}}$) into cell *i* from ion channel *j* is given by:


$$\begin{array}{l} I_{ij}^{\text{(vd)}}={ḡ}_{ij}A^{p}B^{q}\left(V_{i}-E_{ij}\right)f\left[\text{reg}\right] \end{array}$$ (2)

where *A* and *B* are activation and inactivation gating variables, *V*_*i*_ is the membrane potential, *E*_*ij*_ is the reversal potential. The term reg represents the regulation of conductances by second messengers such as ion pools, and is generally a function of concentration such as reg([ion]). The function *f*[reg] determines the effect of increasing or decreasing the factor reg. *q* is either 0 or 1, and *p*≥*q*. The synaptic currents in Equation 1, the formulas for A, B and f [reg] in Equation 2, and the remaining equations used in the model are described in the Appendix. The descriptions and units of the variables appearing in the equations are provided in Table 1, while the remaining variables are dimensionless. The individual components of the SNNAP-based models were then integrated into a compact Python package (Figure 1) called NEURONpyxl, which was developed to use NEURON to numerically solve these equations by automatically generating a network from model parameters alone, taken from an Excel spreadsheet.

**Table 1:** Units of the variables and parameters described in this work, corresponding to the parameters in the Excel spreadsheet interface.

Variable	Description	Units
*I*	Current	Nanoamperes (nA)
*V*	Membrane potential	Millivolts (mV)
*E*	Reversal potential	Millivolts (mV)
*C*_m_	Membrane capacitance	Microfarads (uF)
ḡ	Maximum conductance	Microsiemens (uS)
τ	Time constant	Seconds (s)
[ion]	Ion concentration	Millimolar (mM)
*k*_1_	Ion pool time constant	1/seconds (s^-1)
*k*_2_	Ion pool current scaling constant	Molar/amperes (M A^-1)
*h*	Half-activation voltage	Millivolts (mV)
*s*	Activation slope factor	Millivolts (mV)
γ	Concentration scaling factor	1/millimolar (mM^-1)
*w*	Synaptic weight	Microsiemens (uS)

In NEURON, values such as current, conductance and membrane capacitance are specific, but appropriate unit conversions are done such that input and output values are in absolute units. Any variable or parameter not present in this table is unit-less.

The parameters and equations are specified in the same Excel spreadsheet interface that was developed for SNNAP (Momohara et al., 2022). In this way, any previously developed SNNAP models built with this Excel spreadsheet can be reproduced in NEURONpyxl with the only numerical differences in simulation results arising from integration methods, floating-point error, and noise generation methodologies. SNNAP employs Forward Euler integration, whereas NEURON has several options for integration methods, including the Backward Euler method, Crank-Nicholson method and CVODE solvers (Gardner et al., 2022), but not Forward Euler integration. NEURON also includes variable (adaptive) time-stepping to maintain accuracy while reducing computation time, whereas SNNAP can only integrate using constant time steps. In models where the underlying dynamics are stiff or chaotic, these numerical discrepancies can accumulate over time, potentially leading to different outcomes across simulators, such as different numbers of spikes or different frequency of buccal motor programs (BMPs). Such sensitivity to initial conditions and numerical methods has been observed even in relatively simple models of action potentials (Guttman et al., 1980; Wang, 1993; Canavier et al., 1993; Guckenheimer and Oliva, 2002; Innocenti et al., 2007). Integrating with CVODE solvers in addition to a relaxation period prior to recording state variables established the initial conditions that reduced potential discrepancies. For each simulation, the relaxation period duration was determined by the time at which *dV*/*dt* → 0 for all neurons (default 1 s, ranged up to 10 s).

### 2.2 Parameter search

NEURONpyxl was used to conduct a parameter grid search to locate parameter values in a computational model of the feeding CPG that generated simulated motor patterns with durations of protraction and retraction that matched real feeding behavior under different conditions of mechanical load in Gill and Chiel (2020). We developed an external script to run individual NEURONpyxl simulations. This script searched within the parameter grid to find the parameter combination with the smallest squared difference between the BMP phase durations of the simulations and the empirically measured BMP phase durations in Gill and Chiel (2020). A total of 3600 simulations were run on the Case Western Reserve University Pioneer HPC Cluster, which uses the RedHat Linux 8 operating system. The NEURON simulations run by NEURONpyxl can be run in parallel using Python's multiprocessing package, as long as there is a single process per CPU. Therefore, batches of 16 simulations were run on one node with 16 cores, taking approximately 20 h to complete and reducing the effective per-simulation computation time from over 6 min to 20 s.

## 3 Results

### 3.1 Developing and validating NEURONpyxl

We developed NEURONpyxl by simulating well-understood conductance-based models and comparing them to simulation results from SNNAP or, in the simplest cases, to analytical solutions. In principle, if NEURONpyxl and SNNAP integrate identical systems of ordinary differential equations, then the resulting traces from both simulators should produce identical results, up to numerical errors resulting from integration methods or numerical precision. NEURONpyxl was validated for single neurons, then for simple circuits and finally an entire feeding model CPG (Momohara et al., 2022).

In SNNAP, neurons can be treated as single points. Therefore, to facilitate the SNNAP-to-NEURON conversion, each neuron was represented in NEURON as a single section containing one segment. Neurons with multiple compartments are modeled as two neurons that are tightly coupled with an electrical synapse. The NEURON sections in NEURONpyxl were not implemented with temperature dependence, and were not modeled according to cell morphology. However, NEURONpyxl can readily incorporate complex morphology by incorporating multiple sections and segments.

### 3.2 Leaky integrator neuron model

First, SNNAP and NEURON results were compared for a leaky integrator model neuron stimulated with a current pulse, which has an analytical solution. The change in membrane potential for a leaky integrator is given by:


$$\begin{array}{l} C_{\text{m}}\frac{dV}{dt}=I^{\text{(app)}}-{ḡ}_{\text{leak}}\left(V-V_{\text{leak}}\right) \end{array}$$ (3)

where


$$\begin{array}{l} I^{\text{(app)}}(t)=\{\begin{array}{ll} I_{0}, & t_{0}\leq t<t_{1}\\ 0, & \text{otherwise} \end{array} \end{array}$$

**Figure 2:**
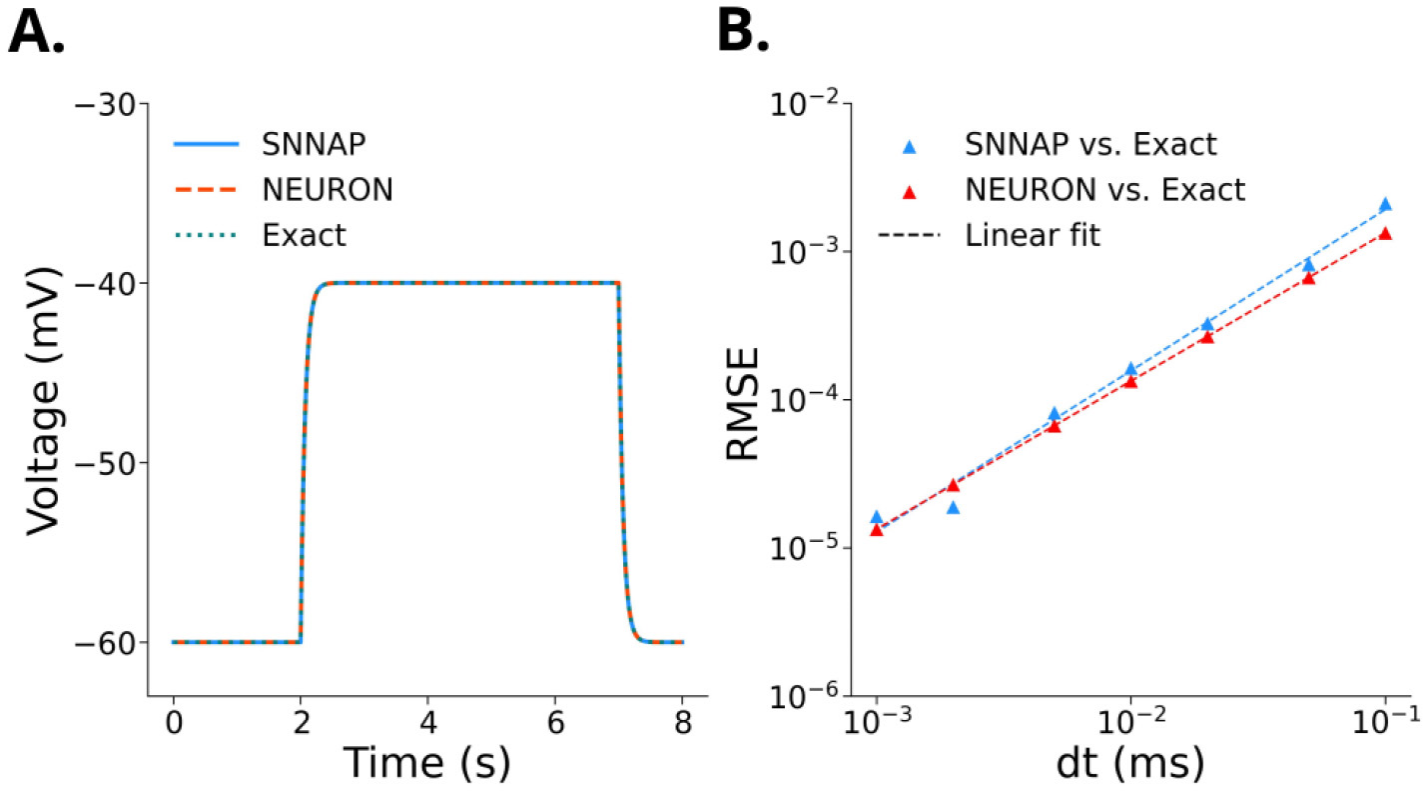
**(A)** Changes in the membrane potential produced by a +2 nA current injection delivered between 2 and 7 s for a model neuron with a leak channel. The exact solution in Equation 4 of Equation 3 is shown, along with numerical computations using SNNAP and NEURON. **(B)** Convergence of the root-mean squared error as a function of timestep when comparing NEURON and SNNAP to the exact solution given by Equation 4.

This differential equation can be solved analytically from the initial condition *V*(0) = *V*_leak_, namely


$$\begin{array}{l} V(t)\;=\left\{ \begin{array}{l} \begin{array}{cc} V_{\text{leak}}, & \;0\leq t<t_{0} \end{array}\\ \begin{array}{cc} V_{\text{leak}}+\frac{I_{0}}{{\bar{g}}_{\text{leak}}}(1-\exp(-(t-t_{0})/\tau )), & t_{0}\leq t<t_{1} \end{array}\\ V_{\text{leak}}+\frac{I_{0}}{{\bar{g}}_{\text{leak}}}(1-\exp(-(t_{1}-t_{0})/\tau ))\\ \begin{array}{cc} \;\exp(-(t-t_{1})/\tau ), & \;t\geq t_{1} \end{array} \end{array}\right. \end{array}$$ (4)

where τ = *C*_m_/ḡ_leak_. The SNNAP model was integrated with Forward Euler integration (Ziv et al., 1994), whereas the NEURON model was integrated using the Crank-Nicholson method (Carnevale and Hines, 2006). These simulations were compared to the exact solution. SNNAP and NEURON produce very similar results compared to the exact solution (Figure 2A). The numerical errors introduced by the integration methods were then compared to the exact solution (Figure 2B). This analysis revealed that Crank-Nicholson integration is more accurate than Forward Euler integration when compared with the exact solution (Figure 2B).

The root-mean-squared errors of the comparisons in Figure 2B converge with a slope of approximately 1 in log space, and the Crank-Nicholson solution converges to the Forward Euler solution for small timesteps. We also compared NEURON's CVODE integration with variable timesteps to the exact solution given by Equation 4, and specified an absolute error tolerance to which the numerical solution converges. By setting the tolerance to 10^−6^, we obtain an RMSE of 10^−6.19^, which is more accurate than both the Forward Euler and Crank-Nicholson integration with constant time steps. Variable time steps are especially useful when considering the balance of computational cost and simulation accuracy and is important to consider in complex and chaotic models. Simulations computed with CVODE solvers (Gardner et al., 2022) resulted in a 2 – 4 fold decrease in computation time when compared to simulations computed with the Crank-Nicholson and Backwards Euler methods.

### 3.3 Spiking neuron model

**Figure 3:**
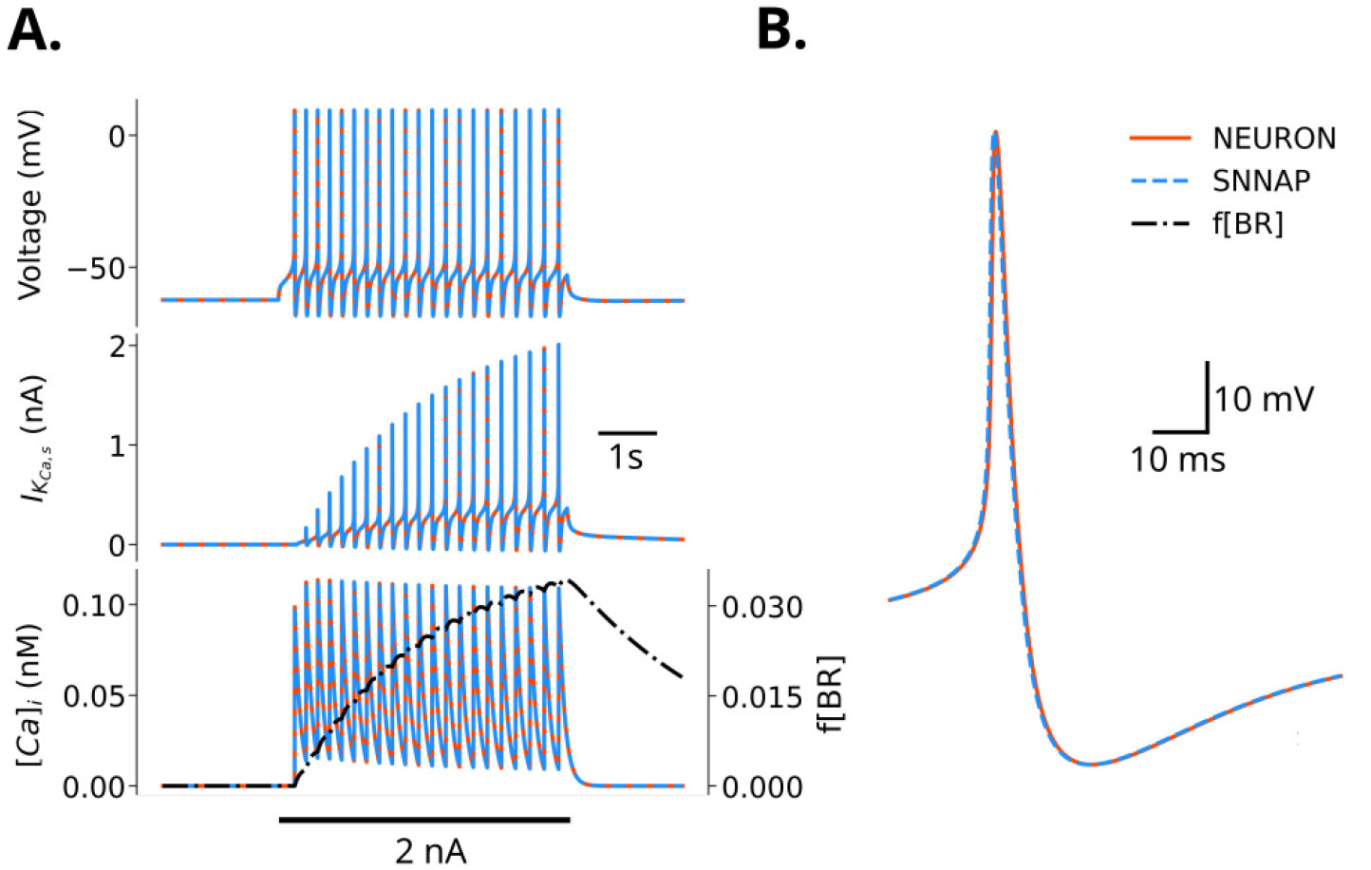
Simulation of the B4 neuron (Momohara et al., 2022). Traces from simulations using SNNAP (blue dashed) and NEURON (orange solid) are overlaid in **(A, B)**. Solid black lines indicate current injection. **(A)** B4 neuron was stimulated with a 2 nA pulse current injection for 5 s. Membrane potential, K_Ca_ current, and Ca^2^+ concentration are shown. The calcium-dependent regulation *f*[*BR*] is shown over the Ca^2^+ concentration trace (black dot-dashed). **(B)** Single action potential from the simulation in **(A)**.

Next, we incorporated voltage-dependent conductances and their regulation by second-messenger pools (e.g., Ca^2^+), mechanisms that enable generation and regulation of spiking. We compared simulation results of the Momohara et al. (2022) B4 neuron model built in SNNAP to the same model created in NEURONpyxl by examining the response to a suprathreshold current injection (Figure 3A). The B4 neuron includes voltage-dependent ion channels that generate action potentials that exhibit spike-rate attenuation. The spike-rate attenuation was mediated by an N-type voltage-dependent Ca^2^+ channel that feeds a Ca^2^+ pool, which in turn regulates Ca^2^+-activated, voltage-dependent potassium channels (K_Ca_). The N-type Ca^2^+ channel is regulated by a negative feedback mechanism mediated by the Ca^2^+ concentration.

Figure 3A shows spike rate attenuation produced by a positive current pulse. As the K_Ca_ current increases, the spike frequency decreases slightly. SNNAP and NEURONpyxl models produce identical results for the Ca^2^+ concentration and voltage-dependent currents such as K_Ca_ (Figure 3A). SNNAP and NEURONpyxl produced identical membrane potentials, even during large voltage changes, such as during an action potential (Figures 3A, B).

### 3.4 Electrical synapses

Electrical synapses are modeled as a resistive coupling (a gap junction) between two neurons *i* and *j*, so the injected current is given by Equation 5.


$$\begin{array}{l} I_{ij}^{\text{(es)}}={ḡ}_{ij}\left(V_{i}-V_{j}\right) \end{array}$$ (5)

**Figure 4:**
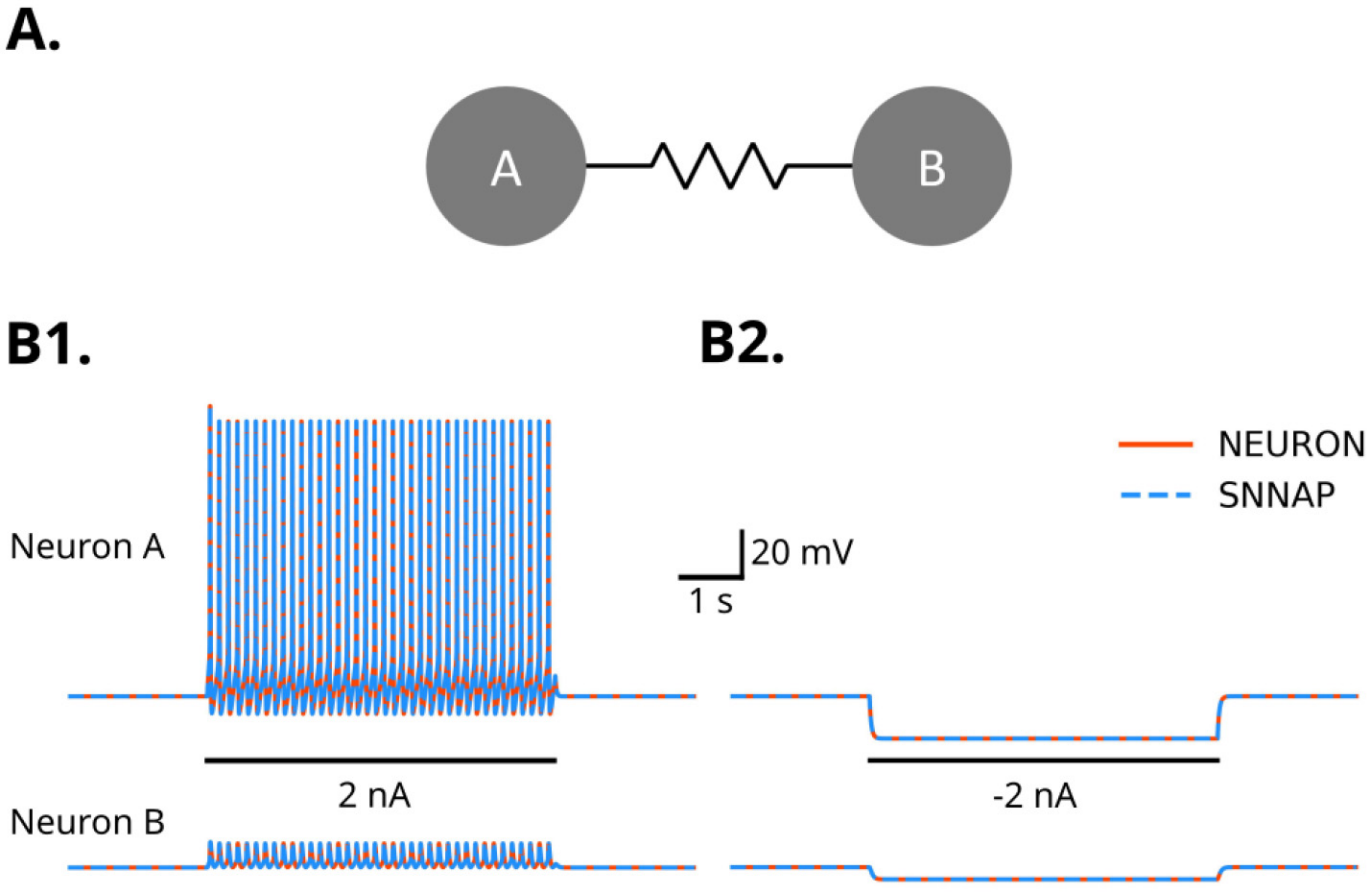
**(A)** Schematic diagram of neurons A and B connected by an electrical synapse (resistor symbol). **(B)** Neuron A is stimulated with 2 nA of depolarizing current for 5 s. The spikes produced in neuron A produce corresponding voltage deflections in neuron B. Traces from simulations using SNNAP (blue dashed) and NEURON (orange solid) traces are overlaid and can be seen to produce equivalent results. Solid black line indicates current injection.

A small circuit of two identical neurons was created with an electrical connection between them. Simulations of this circuit in NEURONpyxl and SNNAP produce identical results (Figure 4). Simultaneous voltage responses in Neuron B can be observed in response to voltage deflections in Neuron A.

### 3.5 Chemical synapses

Chemical synapses are modeled as time-dependent post-synaptic conductances (Tsodyks et al., 2000; Garcia et al., 2022) that are triggered by the occurrence of an action potential in the presynaptic neuron (see List of Equations in Appendix):


$$\begin{array}{l} I_{ij}^{\text{(cs)}}={ḡ}_{ij}M_{1}\left(t\right)M_{2}\left(t,V_{i}\right)\left(V_{i}-E_{ij}\right) \end{array}$$ (6)

where *M*_1_(*t*) defines the time-dependence and *M*_2_(*t, V*_*i*_) defines the voltage-dependence of the synapse (e.g., NMDA receptors) (Ichinose et al., 2003; Clarke and Johnson, 2008), with *V*_*i*_ denoting the voltage of the post-synaptic neuron *i*. *M*_1_(*t*) includes a term *X*(*t*, [*textrmtextion*]) that modulates the activation of the synapse through two possible mechanisms. One mechanism includes a transmitter pool that is regulated by a pool of ions (A10, A11 in Appendix), which is typically used to model synaptic facilitation. The second mechanism includes regulation by presynaptic activity (PSM term, A16–A19 in Appendix), which is used to model synaptic depression. The voltage-dependent activation term in Equation 6, *M*_2_(*t, V*_*i*_), is modeled similarly to voltage-gated ion channel activation (A21, A22 in Appendix).

The threshold for triggering synaptic events was set at 0 mV of the presynaptic neuron. In NEURON, the NetCon object is used to mediate the trigger. Facilitation due to ion pool regulation is implemented in NEURON by creating a pointer to the ion concentration, which is supplied by voltage-gated ion channels. The value of the ion concentration is read into Equation A13 in Appendix, which changes the gain of the synaptic response through Equations A10 and A20 in Appendix.

**Figure 5:**
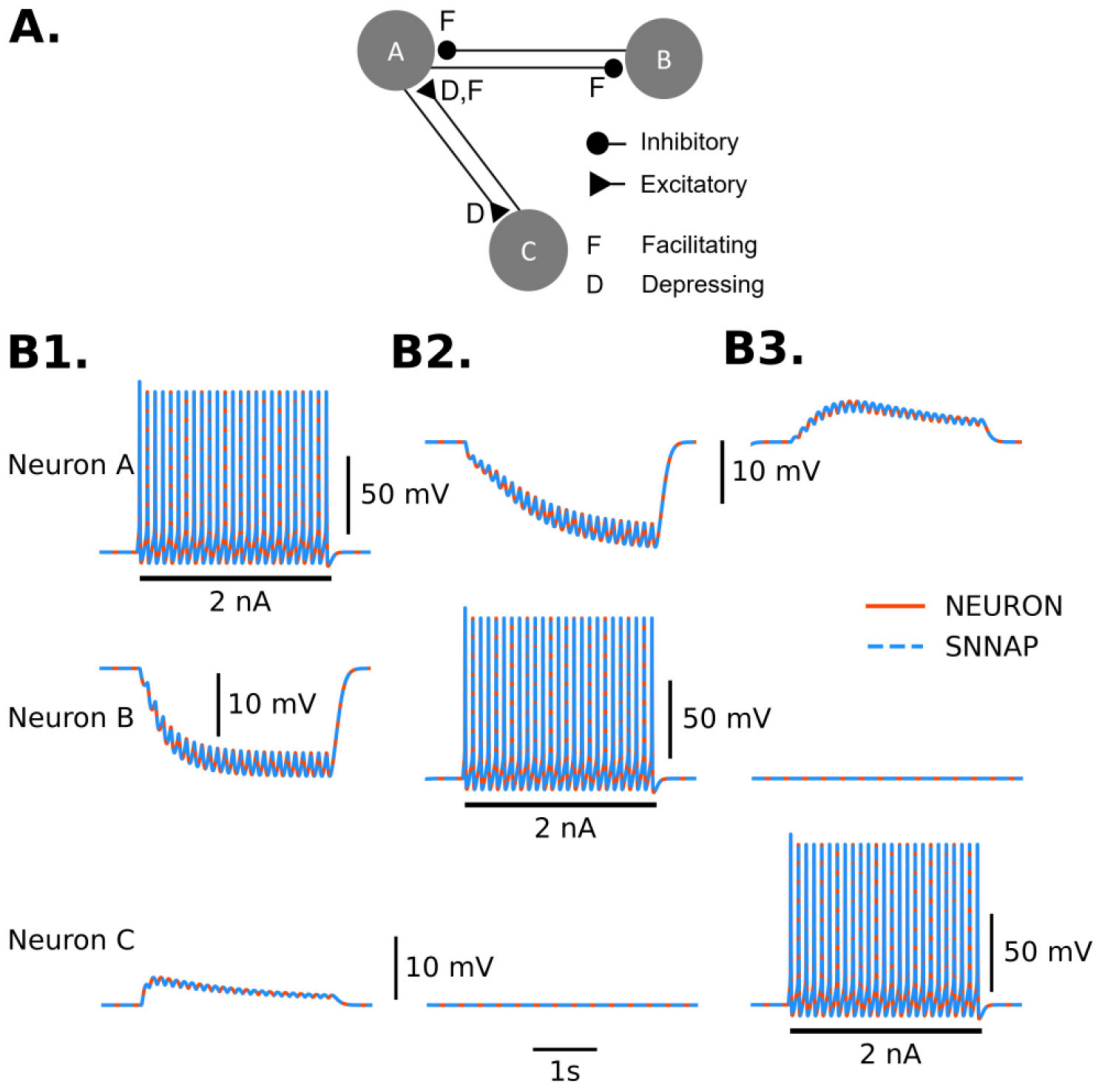
Model network of three identical spiking neurons that includes chemical synaptic connections that are regulated by depression (D) and facilitation (F). **(A)** Diagram of the model network. A → B and B → A are inhibitory synapses that have synaptic facilitation at different rates. A → C is excitatory with synaptic depression. C → A is excitatory and regulated by facilitation and depression. The neurons were held at their resting potential (approximately −70 mV). **(B)** Stimulation of Neuron A **(B1)**, Neuron B **(B2)**, and Neuron C **(B3)**, one at a time, shows the functionality of facilitation and depression. Traces from simulation results using SNNAP (blue dashed) and NEURON (orange solid) are overlaid. Solid black lines indicate current injection.

Model networks that include synaptic connections were implemented in SNNAP and NEURONpyxl (Figure 5). The results from SNNAP and NEURONpyxl both match, which indicates that the synaptic event-handling and models for plasticity are the same as in the SNNAP implementation.

**Figure 6:**
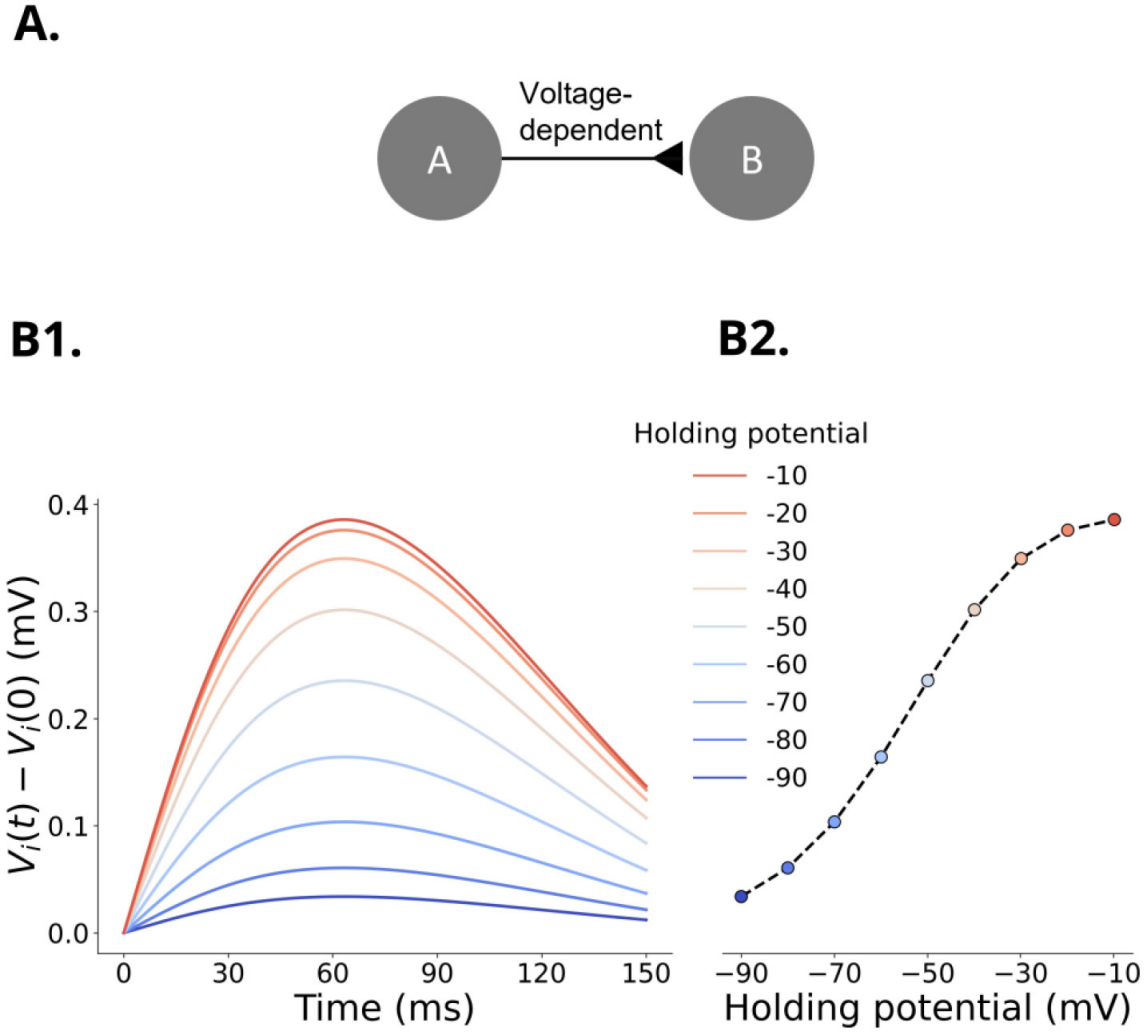
Voltage traces of Neuron B following an action potential in Neuron A while Neuron B was held at membrane potentials ranging from −90 mV to −10 mV. The amplitude of the PSP increased when Neuron B was held at depolarized membrane potentials. **(A)** Neuron A is a simple spiking neuron, and Neuron B is a neuron with a leakage channel only. Neurons A and B are connected by an excitatory synapse with voltage-dependence. **(B)** Simulations of the post-synaptic potential response at different holding potentials of Neuron B. Individual spikes were induced in Neuron A by applying a +1 nA pulse for 150 ms at constant intervals. The voltage of Neuron B is increased by 10 mV by adjusting the holding current. **(B1)** PSPs measured at holding potentials indicated by the color of the trace. **(B2)** Peak post-synaptic potential from simulations in panel B1, which follow a sigmoidal relationship. Results from simulations in SNNAP and NEURON in both **(B1, B2)** are identical. The y-axes for **(B1)** and **(B2)** are the same.

We next implemented voltage-dependent regulation of synaptic connections in SNNAP and NEURON. Spikes in the presynaptic neuron produced post-synaptic potentials (PSP) with amplitudes that depended on the potential of the post-synaptic neuron and these responses were equivalent between SNNAP and NEURONpyxl (Figure 6).

### 3.6 Noise implementation

Firing patterns of neurons are influenced by a variety of noise sources (Faisal et al., 2008). In order to incorporate dynamical variability into simulations, SNNAP implements noise by adjusting the maximal conductance of each ion channel and synapse by a randomly generated number drawn from a normal distribution (Ziv et al., 1994; Baxter and Byrne, 2007). NEURON emphasizes simulation of physiologically-based mechanisms. In NEURONpyxl, we replaced the SNNAP noise mechanism with two independent NetStims (noisy spike train generators) driving a balanced pair of inhibitory and excitatory synapses. Each neuron received its own independent noise sources. Setting the NetStim noise parameter to unity drives the receiving synapse with Poisson spike trains. The “interval” parameter for the inhibitory NetStim should be set to 1/λ_i_, where λ_i_ is the desired rate of inhibitory synaptic input, and similarly for the excitatory NetStim. In practice, we typically set λ_e_ = λ_i_, although this choice is not required. The following equations represent the noisy current injected into each cell.


$$\begin{array}{ll} I^{\left(\text{noise}\right)} & =I_{\text{i}}+I_{\text{e}} \end{array}$$ (7)

$$\begin{array}{ll} I_{\text{i}} & =g_{\text{i}}\left(t\right)\left(V-E_{\text{i}}\right) \end{array}$$ (8)

$$\begin{array}{ll} I_{\text{e}} & =g_{\text{e}}\left(t\right)\left(V-E_{\text{e}}\right) \end{array}$$ (9)

The conductances of the noisy inhibitory and excitatory synapses are given by


$$\begin{array}{ll} \frac{dg_{\text{i}}}{dt} & =-\frac{g_{\text{i}}}{\tau_{\text{i}}}+w_{\text{i}}\underset{j}{\sum }\delta \left(t-T_{j}^{\text{i}}\right) \end{array}$$ (10)

$$\begin{array}{ll} \frac{dg_{\text{e}}}{dt} & =-\frac{g_{\text{e}}}{\tau_{\text{e}}}+w_{\text{e}}\underset{j}{\sum }\delta \left(t-T_{j}^{\text{e}}\right) \end{array}$$ (11)

In order to balance positive and negative fluctuations near the resting potential, we set


$$\begin{array}{l} w_{\text{e}}=w_{\text{i}}\left|\frac{\lambda_{\text{i}}\tau_{\text{i}}\left(E_{\text{e}}-V_{r}\right)}{\lambda_{\text{e}}\tau_{\text{e}}\left(E_{\text{i}}-V_{r}\right)}\right| \end{array}$$ (12)

where *E*_i_ = −90 mV, *E*_e_ = 60 mV, and *V*_*r*_ is the resting potential. The user sets the synaptic weight of the inhibitory synapse to specify the gain of the noise input. The weight of the excitatory synapse for the noise is set according to Equation 12 such that the mean driving force from each synapse is approximately equal. The excitatory and inhibitory Poisson intervals and time constants are equal by default, and the reversal potential in a cell is determined by the voltage of the cell after calling the NEURON function finitialize() (Carnevale and Hines, 2006).

**Figure 7:**
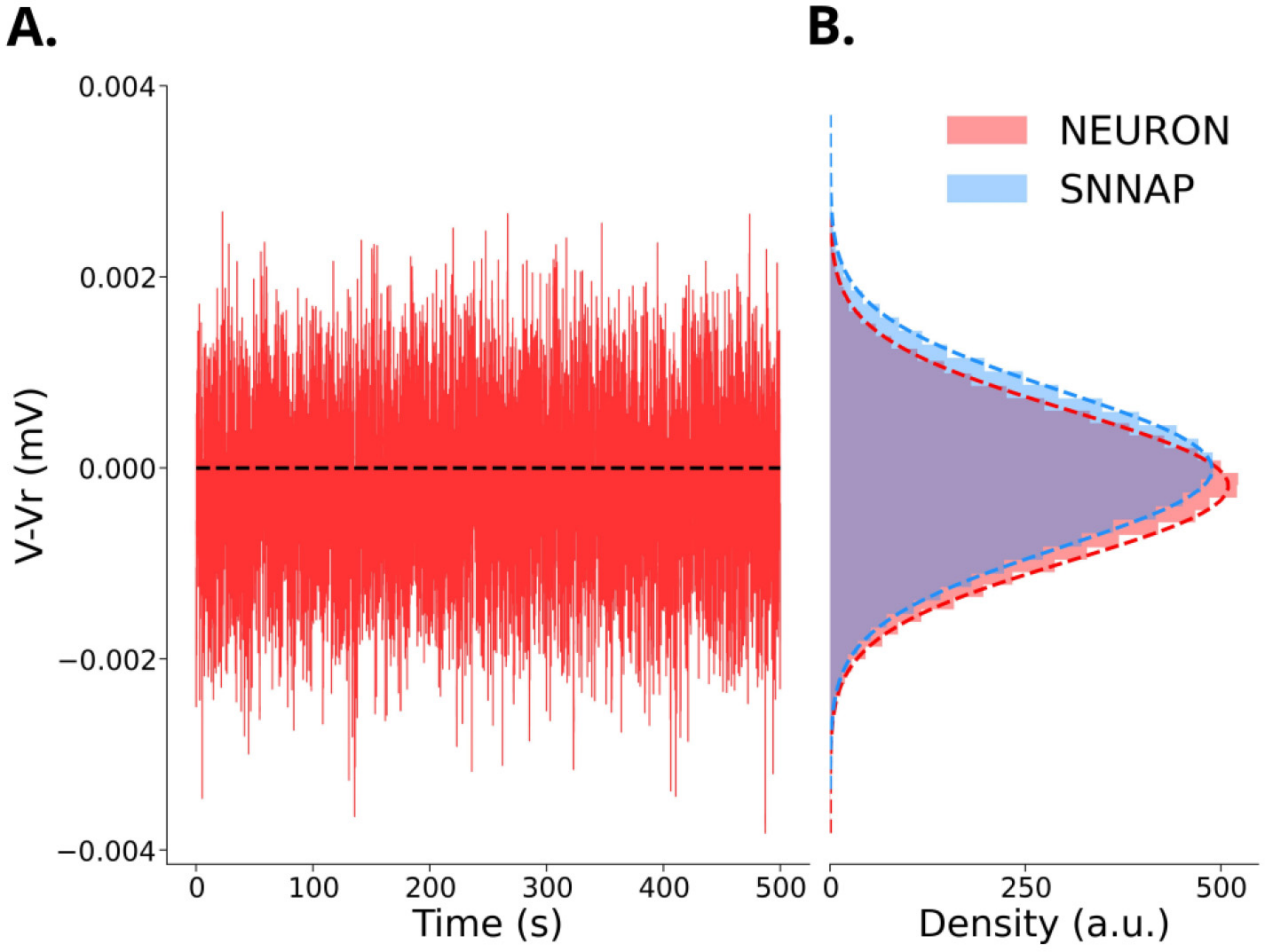
Membrane potential of the B4 neuron (Momohara et al., 2022) in response to noise currents generated by excitatory and inhibitory synapses driven with independent Poisson processes. **(A)** 500 s simulation with NetStim mean interval parameter set to 20 ms, and with inhibitory synaptic weight *w*_i_ = 10 pS, and a synaptic time constant of τ_i_ = τ_e_ = 25 ms. The dashed black line is membrane voltage/current where the weights of the noisy synapses were set to 0. **(B)** Histograms of SNNAP (blue) and NEURON (red) voltage responses each fit with a Gaussian function (red and blue dashed lines). SNNAP simulations were run with ion channel conductances modulated by a random variable *g* = ḡ+*R* updated every 200 timesteps (Δ*t* = 0.5 ms), where $R\sim N\left(\mu =0,\sigma =5\cdot 10^{-4}ḡ\right)$ (Baxter and Byrne, 2007). NEURON simulations were run with the same parameters as **(A)**. The standard deviation was chosen manually so that the distribution of voltage responses was comparable to the NEURON distribution. The bias of the noise, measured as the difference between the mean of the Gaussian fit to the histogram and the resting potential under noiseless conditions, was 0.19 μ*V*. The current bias was +7.6·10^−6^ nA.

Figure 7 shows the response of B4 neuron (Momohara et al., 2022) to synaptic noise. The presence or absence of noise has little effect on membrane potential. Specifically, the voltage bias is 0.2 μ*V* and the current bias is +7.6·10^−6^ nA. We observed that the magnitude of the bias increases gradually with the weights of the inhibitory and excitatory synapses. The results in Figure 7 demonstrate that the noise model described by Equations 7–12 produces a noise profile comparable to that of SNNAP.

### 3.7 Simulating a central pattern generating circuit

**Figure 8:**
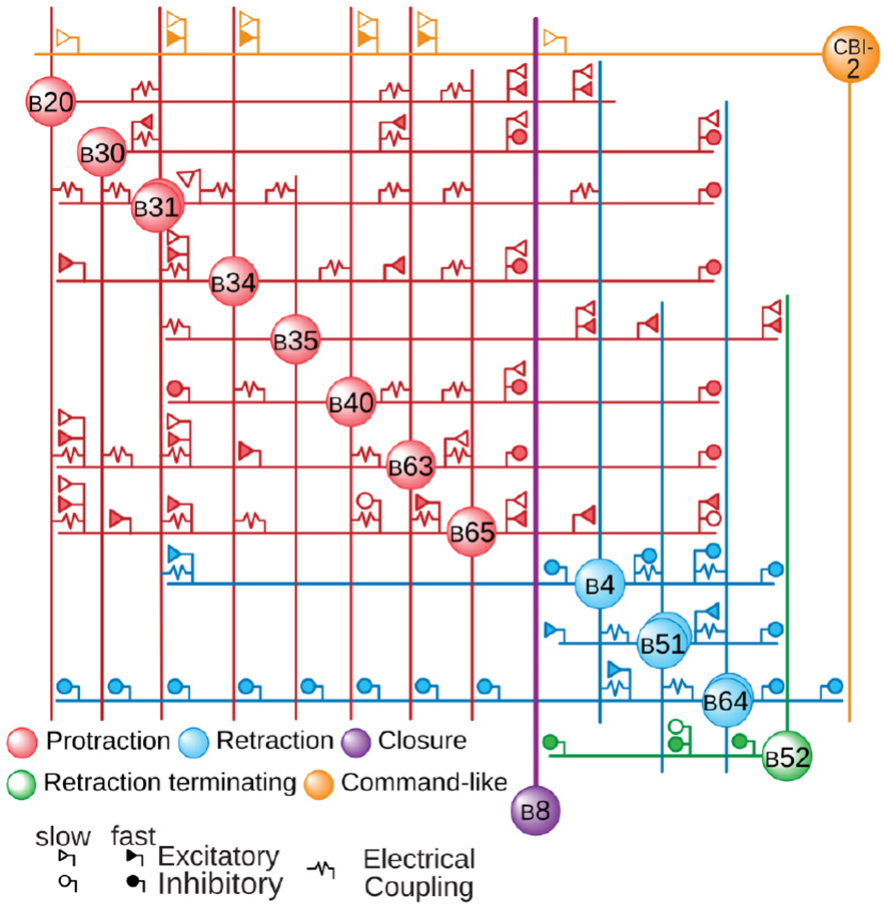
Connectome of the model CPG adapted, with permission, from Figure 5A1 in Momohara et al. (2022). Creative Commons Attribution License 4.0 (CC-BY).

**Figure 9:**
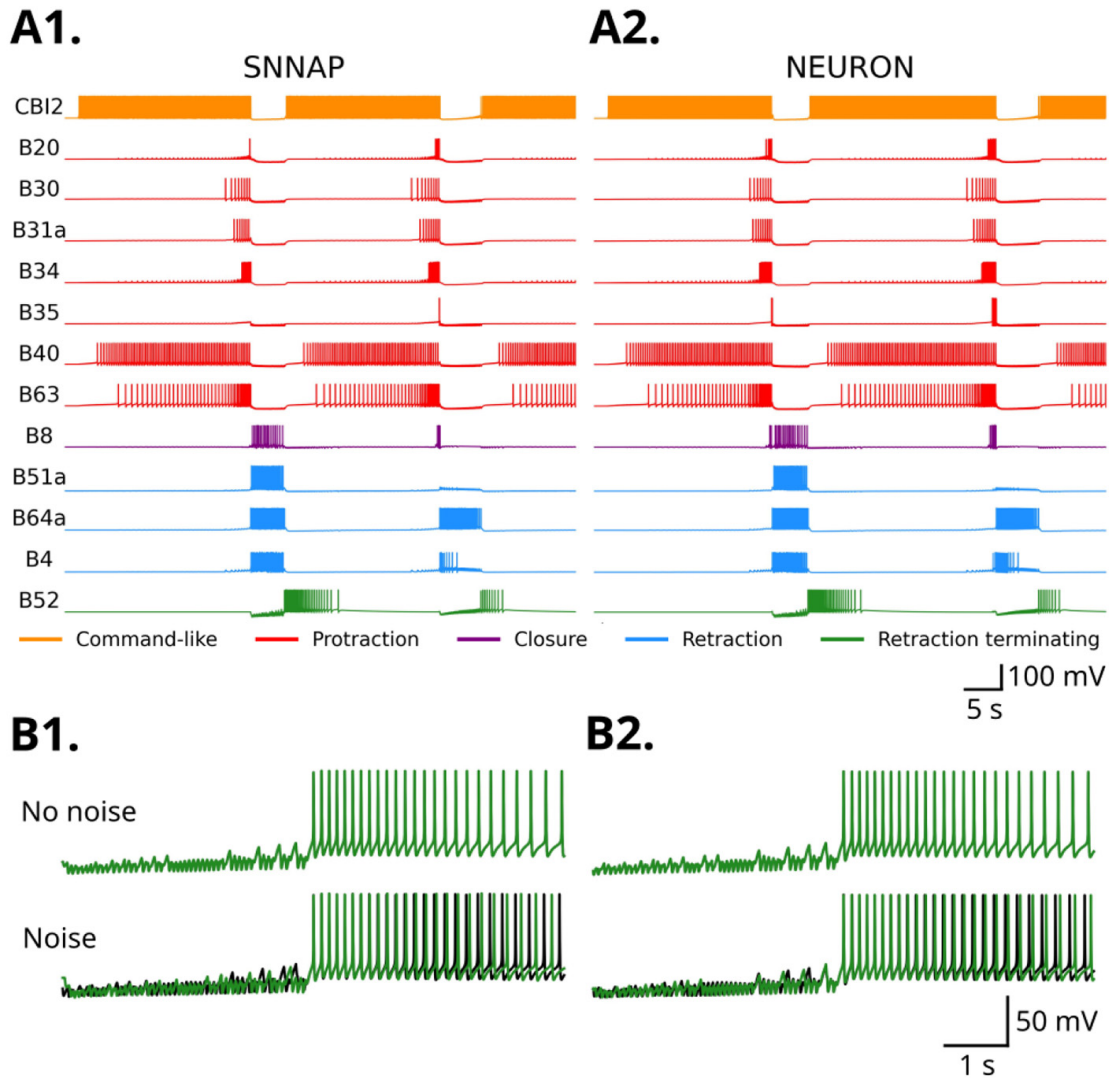
Comparison of SNNAP and NEURON simulations of the full CPG model. **(A)** Simulation of two fictive motor patterns in SNNAP **(A1)** and NEURON **(A2)**. Traces are colored according to the phase at which each neuron is active (protraction is red, retraction is blue) or by its function (command-like is orange, and retraction terminating is green). In SNNAP, noisy conductances were modulated according to a normal distribution with mean 0 μ*S* and standard deviation ḡ/15. In NEURONpyxl, noise was applied at 50 Hz (λ_i_ = λ_e_ = 20 ms) with an inhibitory synaptic weight of $w_{\text{i}}=10^{-5}$ μ*S* and a time constant of τ_i_ = τ_e_ = 25 ms. **(B)** Comparison of two traces from two independent simulations of the B52 neuron with (bottom) and without noise (top) for SNNAP **(B1)** and NEURON **(B2)**. Note that in the absence of noise the overlap between the two runs is complete.

NEURONpyxl was used to reconstruct a model CPG network previously developed by Momohara et al. (2022) (Figure 8) and compared the simulation results of the same network constructed in SNNAP (Figure 9). The Momohara et al. (2022) model (Figure 8) has 14 neurons, three of which (B31, B51, and B64) have both somatic and axonal compartments and the rest have only a single compartment. They are connected through a complex network of 22 electrical synapses and 84 chemical synapses (excitatory and inhibitory), many of which are regulated by facilitation, depression, and voltage-dependence (Figure 8). In both the SNNAP and NEURON simulations, patterned activity was elicited by constant intercellular current injection (+1.87 nA) into the cerebral-to-buccal interneuron 2 (CBI-2) (Perrins and Weiss, 1996). The noise parameters were set to be the same as in Figure 7. We did notice a few discrepancies, notably that there were small increases in activity in B4, B8, B20, and B35 for the model constructed by NEURONpyxl. We also noticed that during the retraction phase, B4 produces 53 spikes in SNNAP and 55 spikes in NEURONpyxl. The ingestion–rejection delay is shorter in SNNAP (22.4 s) compared to NEURON (24.5 s). Despite these small differences, the overall pattern of activity was very similar between the two simulations, indicating that NEURONpyxl correctly reconstructs complex networks generated in SNNAP.

Next, the computation time of SNNAP simulations were compared to NEURONpyxl simulations run on a personal computer (Lenovo ThinkPad L14, Model No. 21C5000UUS, Windows 11). 10 simulations of the Momohara et al. (2022) CPG model constructed using SNNAP (Forwards-Euler with Δ*t* = 7.5 μ*s*) and NEURONpyxl (CVODE integration with variable timesteps) were run for 40 s of simulation time with noise using the same parameters that produce Figure 9. SNNAP simulations of the Momohara et al. (2022) CPG model were manually timed and NEURON simulations were timed using Python's time module. SNNAP simulations took a mean time of 214 ± 16 s and NEURON simulations took 100 ± 5.4 s. NEURONpyxl simulations of the Momohara et al. (2022) CPG model were significantly faster (*t*_18_ = −21.3, *P* ~ 10^−12^).

### 3.8 Parameter search

A parameter grid search was then implemented to locate parameter values in the Momohara et al. (2022) model that generated simulated motor patterns with durations of protraction and retraction matching real feeding behavior under different conditions of mechanical load, as described in Gill and Chiel (2020). As was done in Figure 9, 1.87 nA of current was injected into the CBI-2 neuron with the same noise parameters. The network produced fictive motor patterns that switched between ingestion-like buccal motor patterns and rejection-like patterns. During ingestion-like patterns, B8 activity occurs predominantly during the retraction phase, whereas in rejection-like patterns, B8 activity occurs predominantly during the protraction phase (Morton and Chiel, 1993a,b). The protraction and retraction phases are defined here by spiking activity in B31 (Hurwitz et al., 1996) and B64 (Hurwitz and Susswein, 1996) respectively. We modified the original model such that the simulation only produced ingestion-like patterns by increasing B51 excitability, a neuron with an excitatory synapse to B8 (Plummer and Kirk, 1990; Evans and Cropper, 1998; Nargeot et al., 1999). The excitability of B51 was increased by decreasing the leakage conductance of the axon compartment by 62.5%, and increasing the maximum conductance of the fast sodium channel by 60% in the axon compartment. The increased excitability of B51 increased the excitatory drive to B8 during retraction, thus enabling B8 to spike throughout retraction.

We next chose parameters that affect the phase duration of protraction and retraction. The soma compartment of the neuron defining retraction (B64) contains a persistent sodium channel that elicits an all-or-nothing burst of action potentials lasting several seconds. This burst of activity is terminated by a slow voltage-gated potassium conductance. To modify the duration of retraction, this slow potassium conductance in B64s (ḡ_K_PP_, B64s_) was varied over a linear mesh of 60 values ranging from 0.5 to 1.8 μ*S*. The protraction phase duration, however, is determined by the initiation of B64 activity, which terminates protraction neuron activity through its many inhibitory connections to neurons mediating protraction. B64 is activated by the slow B63 to B64s excitatory connection. B63 is activated, in turn, by the B30 to B63 fast excitatory connection. We found that increasing the B30 to B63 synaptic connection decreased the duration of the protraction phase. Therefore, to modify the duration of protraction, the connection between B30 and B63 (ḡ_B30 → B63, fast_) was varied over 60 values from 1.3 to 4.0 μ*S*. In order to ensure that the phase durations had stabilized, the simulations were run for 120 s of simulation time, and the first buccal motor program was excluded from the analysis.

**Figure 10:**
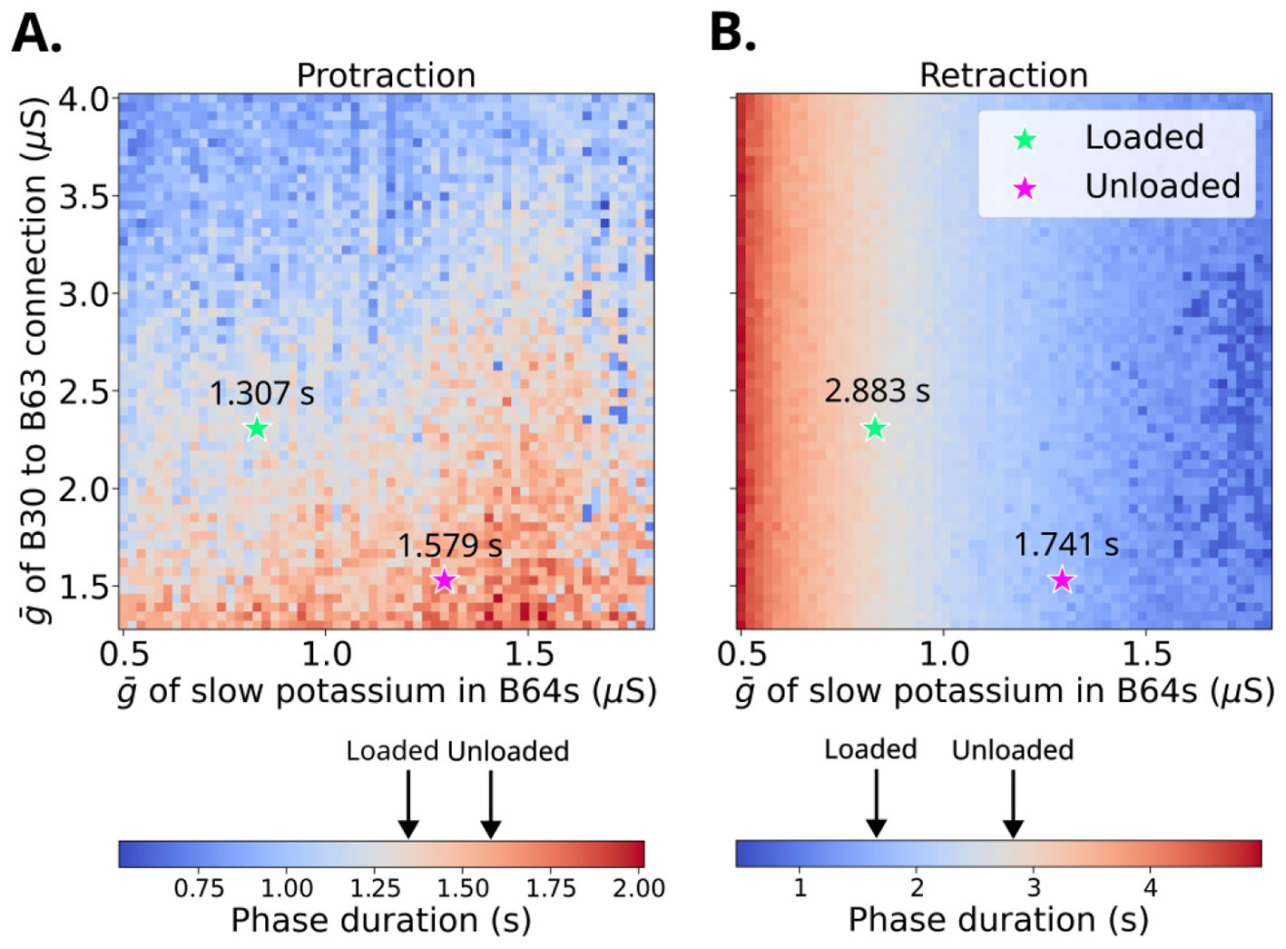
Durations of **(A)** protraction and **(B)** retraction during ingestion as a function of ḡ_K_PP_, B64s_ and ḡ_B30 → B63, fast_. Black arrows on the color bars correspond to the experimentally measured phase durations for loaded and unloaded swallows from Gill and Chiel (2020). The green and magenta stars correspond to the values for ḡ_K_PP_, B64s_ and ḡ_B30 → B63, fast_ in the loaded and unloaded cases which minimize the root-mean-squared error with the empirically-determined phase durations in Gill and Chiel (2020) (labeled black arrows).

**Figure 11:**
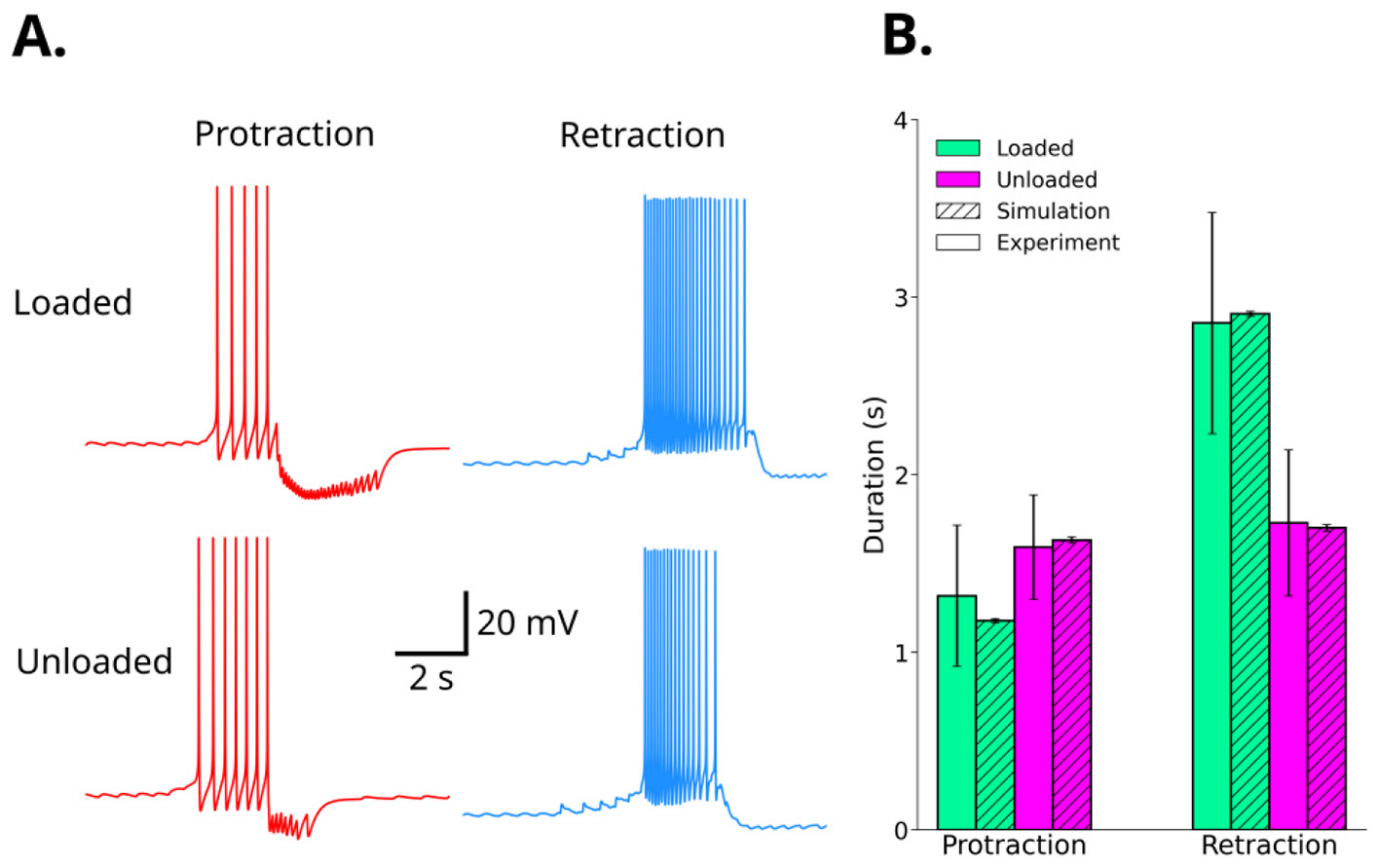
The updated Momohara et al. (2022) model produces motor pattern durations more consistent with Gill and Chiel (2020). **(A)** Example simulations illustrating the protraction (B31a, left) and retraction (B64a, right) phases of a single motor pattern in the loaded (top) and unloaded (bottom) conditions. **(B)** Summary of the protraction and retraction durations for simulations in the loaded and unloaded conditions. Error bars represent the standard error reported in Gill and Chiel (2020) for experimental data, and the standard error of the mean of simulated motor pattern durations for model results.

Heatmaps of the protraction and retraction duration from these simulations are shown in Figure 10. The heatmap of the retraction duration (Figure 10B) has a horizontal gradient, indicating that the retraction duration depends on ḡ_K_PP_, B64s_, but does not depend on ḡ_B30 → B63, fast_. In contrast, the protraction duration (Figure 10A) has a more complex relationship with ḡ_K_PP_, B64s_ and ḡ_B30 → B63, fast_, and appears to depend on both variables. The two points in these heatmaps with the lowest mean-squared error for both retraction and protraction in the loaded and unloaded conditions are indicated by green and purple stars. These optimal parameters are summarized in Table 2. We ran simulations of the model CPG network in which ḡ_K_PP_, B64s_ and ḡ_B30 → B63, fast_ were set to their optimal values for loaded and unloaded conditions (see Table 2). The simulations were run for 120 s. The updated network was observed to be more robust to noise than the network shown in Figure 9, so we increased the noise level (frequency of 200 Hz, $\omega_{\text{e}}=\omega_{\text{i}}=10^{-4}$ μ*S*, and τ_e_ = τ_i_ = 8 s) to test how well the network matched the experimental phase durations. The first motor program was excluded from the analysis, and the mean and standard deviations of the phase durations are calculated for the remaining motor patterns, as was done for the simulations in Figure 10. The voltage traces of the axon compartments of B31 (protraction) and B64 (retraction) are shown in Figure 11 for loaded and unloaded conditions. The phase durations of the optimized model are within the standard error of the experimental data (Figure 11B).

**Table 2:** Conductances (in μ*S*) in the Momohara et al. (2022) CPG network which minimize the Euclidean distance between the computed mean phase duration and those reported in Gill and Chiel (2020).

Condition	ḡ_K_PP_, B64s_	ḡ_B30 → B63, fast_
Original	0.35	0.25
Unloaded	1.293	1.529
Loaded	0.831	2.307

We conclude that our optimized model produces stable ingestion-like patterns with mean durations of protraction and retraction that match the experimental data from Gill and Chiel (2020), even in the presence of noise.

## 4 Discussion

We developed NEURONpyxl to create realistic computational models and validated these models by comparing the simulation results to those generated by the SNNAP simulation platform, analytic solutions, and empirical data. We modified the Momohara et al. (2022) network so that it robustly produces ingestion buccal motor patterns even in the presence of noise. Using a parameter sweep, we found two parameter value combinations (Table 2) that produce phase durations of protraction and retraction that match empirical values from loaded and unloaded conditions Gill and Chiel (2020), which are robust in the presence of noise. The parameter mesh for protraction duration in Figure 10 appeared more noisy compared to the retraction heatmap, which could be due to a greater number of connections and recurrent connectivity in the protraction circuitry leading to more sensitivity to parameter changes. Systematic exploration across a wide parameter range allowed us to identify the parameter combinations, listed in Table 2, that produced robust and physiologically relevant behavior.

There was a small discrepancy in number of spikes generated by SNNAP and NEURONpyxl for simulations of the feeding CPG. We attribute these differences to solver accuracy: SNNAP uses Forward Euler, whereas NEURON uses adaptive higher-order CVODE methods. In this highly non-linear network, small numerical differences propagate and lead to measurable changes in circuit-level outputs such as spike counts and motor pattern frequency. Thus, the adaptive higher-order CVODE method of NEURON provide a more realistic output of the Momohara et al. (2022) network than the SNNAP results.

We found that modifying both parameters were necessary to match the behaviors under the two conditions (i.e., loaded and unloaded), suggesting that parameters need to be varied in a coordinated fashion. Similarly, it may be necessary for modulatory substances to induce variations in multiple parameters in order to express their effects (Brezina and Weiss, 2000). We only explored the effects of two of the many parameters in the model. As suggested by the work of Prinz et al. (2004) and Marder and Taylor (2011), other combinations of parameters may also yield similar results. Creating ensembles of models with varying parameters would be important to understand the degree of redundancy in this model system.

A parameter sweep can generate testable hypotheses about the way sensory feedback may stabilize a motor pattern generator. Many motor behaviors involve repetitive execution of a sequence of actions that alternate between a powerstroke, in which an animal attempts to perform mechanical work on an external load, and a recovery phase, in which an animal repositions itself in order to initiate another power stroke (Yu and Thomas, 2024). In bipedal walking, the powerstroke for a given limb corresponds to the stance phase, while the swing phase is the recovery (Spardy et al., 2011a,b). During *Aplysia* ingestive activity, retraction (while grasping seaweed) comprises the powerstroke, and protraction is the recovery phase. Along with biomechanics, sensory feedback from the motor system to the CPG can play an important role in stabilizing rhythmic behavior and facilitating robust adaptation to changes in external load (Kuo, 2002; Pearson, 2008; Yu and Thomas, 2021; Wang et al., 2022). Yet because sensory information is typically carried by afferent axons that are much smaller in diameter than efferent motor neurons, sensory signals are difficult to measure experimentally. Consequently, less is known about the anatomical and functional connectivity of sensory feedback pathways than efferent motor pathways. Because scientific computing tools such as Python make it straightforward to perform parameter sweeps for optimizing model conductances to match experimental data collected under varying mechanical loads, the methods presented here have the potential to illuminate mechanisms of sensory feedback in the *Aplysia* feeding motor system.

More broadly, our study provided a flexible framework to perform complex simulations of conductance-based models and CPGs given model parameters alone, placing NEURONpyxl uniquely among existing neural simulators. NEURONpyxl was designed for parameter-centric model specification rather than the construction of large-scale standardized networks, which may be efficiently constructed with tools such as NetPyNE and PyNN. The SONATA format is well-suited for constructing models to be run using multiple different compatible simulators. Simulators such as Brian2 provide a standalone modeling environment within the Python API, separate from the NEURON simulation framework. Table 3 on page 26 compares the several widely used modern neural simulator frameworks.

**Table 3:** Comparison of commonly used neural simulation frameworks, including NEURONpyxl.

Framework	Intended usage	Scale	Strengths
*NetPyNE*	High-level construction and analysis of NEURON network models in Python API	Medium to large-scale networks	Utilizes parallel NEURON; analysis tools, batch simulations and parameter exploration
*Brian2*	Flexible equation-based neural modeling in Python	Single neurons to large abstract networks	Standalone simulator; concise mathematical model specification; spans multiple scales
*PyNN*	Simulator-independent network specification	Medium to large-scale networks	Unified API for multiple simulators (e.g., NEURON, NEST); high-level user interface
*SONATA*	Standardized large-scale model description format	Large-scale distributed simulations	Cross-simulator compatibility; efficient storage of connectivity and activity data
*NEURONpyxl*	Parameter-centric model description emphasizing biophysical modeling	Single neurons to small/medium networks	Explicit mapping between pre-defined parameters and ODE systems; rapid generation of related models; minimal programming prerequisite; easily integrated with other custom NEURON models

Because NEURONpyxl models are seamlessly integrated into the Python programming environment, this tool allows for additional customizations, models and data analyses that can interact immediately with simulation results. By lowering the barrier to running conductance-based models without directly requiring programming, NEURONpyxl broadens accessibility to biophysical computational modeling. At the same time, its native integration with the Python ecosystem creates opportunities for implementing data-driven workflows, including parameter inference, optimization, and data assimilation, which was considered outside the scope of this work.

Together, these features position NEURONpyxl as a practical tool for both exploratory modeling and extensive studies on neural dynamics.

## Data Availability

The datasets presented in this study can be found in online repositories. The names of the repository/repositories and accession number(s) can be found below: https://doi.org/10.5061/dryad.1c59zw488, Dryad; https://github.com/CWRUChielLab/neuronpyxl and https://github.com/CWRUChielLab/NEURONpyxl-2026-figures, GitHub.

## References

[B1] Bässler U. (1986). On the definition of central pattern generator and its sensory control. Biol. Cybern. 54, 65–69. doi: 10.1007/BF00337116

[B2] Bässler U. Büschges A. (1998). Pattern generation for stick insect walking movements—multisensory control of a locomotor program. Brain Res. Rev. 27, 65–88. doi: 10.1016/S0165-0173(98)00006-X 9639677

[B3] Baxter D. A. Byrne J. H. (2006). Feeding behavior of *Aplysia*: a model system for comparing cellular mechanisms of classical and operant conditioning. Learn.Mem. 13, 669–680. doi: 10.1101/lm.339206 17142299

[B4] Baxter D. A. Byrne J. H. (2007). “Simulator for neural networks and action potentials,” in Neuroinformatics, eds. C. J. Crasto and S. H. Koslow (Totowa, NJ: Humana Press), 127–154. doi: 10.1007/978-1-59745-520-6_8 18368364

[B5] Baxter D. A. Canavier C. C. Clark J. W. Byrne J. H. (1999). Computational model of the serotonergic modulation of sensory neurons in *Aplysia. J. Neurophysiol*. 82, 2914–2935. doi: 10.1152/jn.1999.82.6.2914 10601429

[B6] Bower J. M. Beeman D. (2012). The Book of GENESIS: Exploring Realistic Neural Models with the GEneral NEural SImulation System. New York, NY: Springer-Verlag, Inc.

[B7] Brezina V. Weiss K. R. (2000). The neuromuscular transform constrains the production of functional rhythmic behaviors. J. Neurophysiol. 83, 232–259. doi: 10.1152/jn.2000.83.1.232 10634869

[B8] Burrell B. D. Crisp K. M. (2008). Serotonergic modulation of afterhyperpolarization in a neuron that contributes to learning in the leech. J. Neurophysiol. 99, 605–616. doi: 10.1152/jn.00989.2007 18046001

[B9] Butera R. J. Rinzel J. Smith J. C. (1999). Models of respiratory rhythm generation in the pre-Bötzinger complex. I. Bursting pacemaker neurons. J. Neurophysiol. 82, 382–397. doi: 10.1152/jn.1999.82.1.382 10400966

[B10] Cai Y. Baxter D. A. Crow T. (2003). Computational study of enhanced excitability in Hermissenda: membrane conductances modulated by 5-HT. J. Comput. Neurosci. 15, 105–121. doi: 10.1023/A:1024479020420 12843698

[B11] Canavier C. C. Baxter D. A. Clark J. W. Byrne J. H. (1993). Nonlinear dynamics in a model neuron provide a novel mechanism for transient synaptic inputs to produce long-term alterations of postsynaptic activity. J. Neurophysiol. 69, 2252–2257. doi: 10.1152/jn.1993.69.6.2252 8350142

[B12] Carnevale N. T. Hines M. L. (2006). The NEURON Book. Cambridge: Cambridge University Press. doi: 10.1017/CBO9780511541612

[B13] Church P. J. Lloyd P. E. (1994). Activity of multiple identified motor neurons recorded intracellularly during evoked feedinglike motor programs in Aplysia. J. Neurophysiol. 72, 1794–1809. doi: 10.1152/jn.1994.72.4.1794 7823102

[B14] Clarke R. J. Johnson J. W. (2008). Voltage-dependent gating of NR1/2B NMDA receptors. J. Physiol. 586, 5727–5741. doi: 10.1113/jphysiol.2008.160622 18936081 PMC2655412

[B15] Costa R. M. Baxter D. A. Byrne J. H. (2020). Computational model of the distributed representation of operant reward memory: combinatoric engagement of intrinsic and synaptic plasticity mechanisms. Learn. Mem.27, 236–249. doi: 10.1101/lm.051367.120 32414941 PMC7233148

[B16] Cropper E. C. Evans C. G. Hurwitz I. Jing J. Proekt A. Romero A. . (2004). Feeding neural networks in the mollusc Aplysia. Neurosignals 13, 70–86. doi: 10.1159/000076159 15004426

[B17] Dai K. Hernando J. Billeh Y. N. Gratiy S. L. Planas J. Davison A. P. . (2020). The sonata data format for efficient description of large-scale network models. PLoS Comput. Biol. 16:e1007696. doi: 10.1371/journal.pcbi.1007696 32092054 PMC7058350

[B18] Davison A. P. Brüderle D. Eppler J. M. Kremkow J. Muller E. Pecevski D. . (2009). PyNN: a common interface for neuronal network simulators. Front. Neuroinform. 2:11. doi: 10.3389/neuro.11.011.2008 19194529 PMC2634533

[B19] Diekman C. O. Thomas P. J. Wilson C. G. (2017). Eupnea, tachypnea, and autoresuscitation in a closed-loop respiratory control model. J. Neurophysiol. 118, 2194–2215. doi: 10.1152/jn.00170.2017 28724778 PMC5626889

[B20] Diekman C. O. Thomas P. J. Wilson C. G. (2024). COVID-19 and silent hypoxemia in a minimal closed-loop model of the respiratory rhythm generator. Biol. Cybern. 118, 145–163. doi: 10.1007/s00422-024-00989-w 38884785 PMC11289179

[B21] Dura-Bernal S. Suter B. A. Gleeson P. Cantarelli M. Quintana A. Rodriguez F. . (2019). NetPyNE, a tool for data-driven multiscale modeling of brain circuits. eLife 8:e44494. doi: 10.7554/eLife.44494 31025934 PMC6534378

[B22] Evans C. G. Cropper E. C. (1998). Proprioceptive input to feeding motor programs in *Aplysia. J. Neurosci*. 18, 8016–8031. doi: 10.1523/JNEUROSCI.18-19-08016.1998 PMC6793013 9742168

[B23] Faisal A. A. Selen L. P. J. Wolpert D. M. (2008). Noise in the nervous system. Nat. Rev. Neurosci. 9, 292–303. doi: 10.1038/nrn2258 18319728 PMC2631351

[B24] Fietkiewicz C. McDougal R. A. Corrales Marco D. Chiel H. J. Thomas P. J. (2023). Tutorial: using NEURON for neuromechanical simulations. Front. Comput. Neurosci. 17:1143323. doi: 10.3389/fncom.2023.1143323 37583894 PMC10424731

[B25] Garcia J. W. Bartol T. M. Sejnowski T. J. (2022). Multiscale modeling of presynaptic dynamics from molecular to mesoscale. PLoS Comput. Biol. 18:e1010068. doi: 10.1371/journal.pcbi.1010068 35533198 PMC9119629

[B26] Gardner D. J. Reynolds D. R. Woodward C. S. Balos C. J. (2022). Enabling new flexibility in the SUNDIALS suite of nonlinear and differential/algebraic equation solvers. ACM Trans. Math. Softw. 48, 1–24. doi: 10.1145/3539801

[B27] Gewaltig M.-O. Diesmann M. (2007). NEST (NEural Simulation Tool). Scholarpedia 2:1430. doi: 10.4249/scholarpedia.1430

[B28] Gill J. P. Chiel H. J. (2020). Rapid adaptation to changing mechanical load by ordered recruitment of identified motor neurons. eNeuro 7:ENEURO.0016-20.2020. doi: 10.1523/ENEURO.0016-20.2020 PMC7242813 32332081

[B29] Guckenheimer J. Oliva R. A. (2002). Chaos in the Hodgkin-Huxley model. SIAM J. Appl. Dyn. Syst. 1, 105–114. doi: 10.1137/S1111111101394040

[B30] Guertin P. A. (2013). Central pattern generator for locomotion: anatomical, physiological, and pathophysiological considerations. Front. Neurol. 3:183. doi: 10.3389/fneur.2012.00183 23403923 PMC3567435

[B31] Guttman R. Lewis S. Rinzel J. (1980). Control of repetitive firing in squid axon membrane as a model for a neuroneoscillator. J. Physiol. 305, 377–395. doi: 10.1113/jphysiol.1980.sp013370 7441560 PMC1282979

[B32] Hurwitz I. Neustadter D. Morton D. W. Chiel H. J. Susswein A. J. (1996). Activity patterns of the B31/B32 pattern initiators innervating the I2 muscle of the buccal mass during normal feeding movements in *Aplysia californica. J. Neurophysiol*. 75, 1309–1326. doi: 10.1152/jn.1996.75.4.1309 8727380

[B33] Hurwitz I. Susswein A. J. (1996). B64, a newly identified central pattern generator element producing a phase switch from protraction to retraction in buccal motor programs of *Aplysia californica. J. Neurophysiol*. 75, 1327–1344. doi: 10.1152/jn.1996.75.4.1327 8727381

[B34] Hussain M. A. Grill W. M. Pelot N. A. (2024). Highly efficient modeling and optimization of neural fiber responses to electrical stimulation. Nat. Commun. 15:7597. doi: 10.1038/s41467-024-51709-8 39217179 PMC11365978

[B35] Ichinose T. Yu S. Wang X. Q. Yu S. P. (2003). Ca^2^+-independent, but voltage- and activity-dependent regulation of the NMDA receptor outward K+ current in mouse cortical neurons. J. Physiol. 551, 403–417. doi: 10.1113/jphysiol.2003.041723 12860921 PMC2343239

[B36] Innocenti G. Morelli A. Genesio R. Torcini A. (2007). Dynamical phases of the Hindmarsh-Rose neuronal model: studies of the transition from bursting to spiking chaos. Chaos: Interdiscipl. J. Nonlin. Sci. 17:043128. doi: 10.1063/1.2818153 18163792

[B37] Kuo A. D. (2002). The relative roles of feedforward and feedback in the control of rhythmic movements. Motor Control 6, 129–145. doi: 10.1123/mcj.6.2.129 12122223

[B38] Kupfermann I. (1974). Feeding behavior in textitAplysia: a simple system for the study of motivation. Behav. Biol. 10, 1–26. doi: 10.1016/S0091-6773(74)91644-7 4815142

[B39] Lamb D. G. Calabrese R. L. (2011). Neural circuits controlling behavior and autonomic functions in medicinal leeches. Neural Syst. Circuits 1:13. doi: 10.1186/2042-1001-1-13 22329853 PMC3278399

[B40] Li Y. Webster-Wood V. A. Gill J. P. Sutton G. P. Chiel H. J. Quinn R. D. (2024). A computational neural model that incorporates both intrinsic dynamics and sensory feedback in the *Aplysia* feeding network. Biol. Cybern. 118, 187–213. doi: 10.1007/s00422-024-00991-2 38769189 PMC11289348

[B41] Lyttle D. N. Gill J. P. Shaw K. M. Thomas P. J. Chiel H. J. (2017). Robustness, flexibility, and sensitivity in a multifunctional motor control model. Biol. Cybern. 111, 25–47. doi: 10.1007/s00422-016-0704-8 28004255 PMC5326633

[B42] Manor Y. Bose A. Booth V. Nadim F. (2003). Contribution of synaptic depression to phase maintenance in a model rhythmic network. J. Neurophysiol. 90, 3513–3528. doi: 10.1152/jn.00411.2003 12815020

[B43] Marder E. Taylor A. L. (2011). Multiple models to capture the variability in biological neurons and networks. Nat. Neurosci. 14, 133–138. doi: 10.1038/nn.2735 21270780 PMC3686573

[B44] Momohara Y. Neveu C. L. Chen H.-M. Baxter D. A. Byrne J. H. (2022). Specific plasticity loci and their synergism mediate operant conditioning. J. Neurosci. 42, 1211–1223. doi: 10.1523/JNEUROSCI.1722-21.2021 34992131 PMC8883845

[B45] Morton D. W. Chiel H. J. (1993a). *In vivo* buccal nerve activity that distinguishes ingestion from rejection can be used to predict behavioral transitions in Aplysia. J. Comparat. Physiol. A 172, 17–32. doi: 10.1007/BF00214712 8445578

[B46] Morton D. W. Chiel H. J. (1993b). The timing of activity in motor neurons that produce radula movements distinguishes ingestion from rejection in Aplysia. J. Comparat. Physiol. A 173, 519–536. doi: 10.1007/BF00197761 8263840

[B47] Nargeot R. Baxter D. A. Byrne J. H. (1999). *In vitro* analog of operant conditioning in *Aplysia*. II. Modifications of the functional dynamics of an identified neuron contribute to motor pattern selection. J. Neurosci. 19, 2261–2272. doi: 10.1523/JNEUROSCI.19-06-02261.1999 10066277 PMC6782536

[B48] Neveu C. L. Smolen P. Baxter D. A. Byrne J. H. (2023). Voltage- and calcium-gated membrane currents tune the plateau potential properties of multiple neuron types. J. Neurosci. 43, 7601–7615. doi: 10.1523/JNEUROSCI.0789-23.2023 37699717 PMC10634553

[B49] Orozco Valero A. Rodríguez-González V. Montobbio N. Casal M. A. Tlaie A. Pelayo F. . (2025). A Python toolbox for neural circuit parameter inference. npj Syst. Biol. Applic. 11:45. doi: 10.1038/s41540-025-00527-9 PMC12064716 40346107

[B50] Pearson K. (2008). Role of sensory feedback in the control of stance duration in walking cats. Brain Res. Rev. 57, 222–227. doi: 10.1016/j.brainresrev.2007.06.014 17761295

[B51] Perrins R. Weiss K. R. (1996). A cerebral central pattern generator in *Aplysia* and its connections with buccal feeding circuitry. J. Neurosci. 16, 7030–7045. doi: 10.1523/JNEUROSCI.16-21-07030.1996 8824339 PMC6579257

[B52] Plummer M. R. Kirk M. D. (1990). Premotor neurons B51 and B52 in the buccal ganglia of *Aplysia californica*: synaptic connections, effects on ongoing motor rhythms, and peptide modulation. J. Neurophysiol. 63, 539–558. doi: 10.1152/jn.1990.63.3.539 2329360

[B53] Prinz A. A. Bucher D. Marder E. (2004). Similar network activity from disparate circuit parameters. Nat. Neurosci. 7, 1345–1352. doi: 10.1038/nn1352 15558066

[B54] Sigvardt K. A. Williams T. L. (1992). Models of central pattern generators as oscillators: the lamprey locomotor CPG. Semin. Neurosci. 4, 37–46. doi: 10.1016/1044-5765(92)90032-W

[B55] Skinner F. K. Kopell N. Marder E. (1994). Mechanisms for oscillation and frequency control in reciprocally inhibitory model neural networks. J. Comput. Neurosci. 1, 69–87. doi: 10.1007/BF00962719 8792226

[B56] Spardy L. E. Markin S. N. Shevtsova N. A. Prilutsky B. I. Rybak I. A. Rubin J. E. (2011a). A dynamical systems analysis of afferent control in a neuromechanical model of locomotion: I. Rhythm generation. J. Neural Engi. 8:065003. doi: 10.1088/1741-2560/8/6/065003 PMC3422643 22058274

[B57] Spardy L. E. Markin S. N. Shevtsova N. A. Prilutsky B. I. Rybak I. A. Rubin J. E. (2011b). A dynamical systems analysis of afferent control in a neuromechanical model of locomotion: II. Phase asymmetry. J. Neural Eng. 8:065004. doi: 10.1088/1741-2560/8/6/065004 22058275 PMC3422648

[B58] Stimberg M. Brette R. Goodman D. F. (2019). Brian 2, an intuitive and efficient neural simulator. Elife 8:e47314. doi: 10.7554/eLife.47314 31429824 PMC6786860

[B59] Sukhnandan R. Chen Q. Shen J. Pao S. Huan Y. Sutton G. P. . (2024). Full Hill-type muscle model of the I1/I3 retractor muscle complex in *Aplysia californica. Biol. Cybern*. 118, 165–185. doi: 10.1007/s00422-024-00990-3 PMC11289039 38922432

[B60] Susswein A. Byrne J. (1988). Identification and characterization of neurons initiating patterned neural activity in the buccal ganglia of aplysia. J. Neurosci. 8, 2049–2061. doi: 10.1523/JNEUROSCI.08-06-02049.1988 3385489 PMC6569330

[B61] Sutton G. P. Macknin J. B. Gartman S. S. Sunny G. P. Beer R. D. Crago P. E. . (2004). Passive hinge forces in the feeding apparatus of *Aplysia* aid retraction during biting but not during swallowing. J. Comparat. Physiol. A 190, 501–514. doi: 10.1007/s00359-004-0517-4 15098133

[B62] Tsodyks t. Uziel A. Markram H. (2000). t synchrony generation in recurrent networks with frequency-dependent synapses. J. Neurosci. 20, RC50–RC50. doi: 10.1523/JNEUROSCI.20-01-j0003.2000 PMC6774142 10627627

[B63] Wang X.-J. Rinzel J. (1992). Alternating and synchronous rhythms in reciprocally inhibitory model neurons. Neural Comput. 4, 84–97. doi: 10.1162/neco.1992.4.1.84

[B64] Wang X. J. (1993). Genesis of bursting oscillations in the Hindmarsh-Rose model and homoclinicity to a chaotic saddle. Physica D: Nonlin. Phenomena 62, 263–274. doi: 10.1016/0167-2789(93)90286-A

[B65] Wang Y. Gill J. P. Chiel H. J. Thomas P. J. (2022). Variational and phase response analysis for limit cycles with hard boundaries, with applications to neuromechanical control problems. Biol. Cybern. 116, 687–710. doi: 10.1007/s00422-022-00951-8 36396795 PMC9691512

[B66] Webster-Wood V. A. Gill J. P. Thomas P. J. Chiel H. J. (2020). Control for multifunctionality: bioinspired control based on feeding in *Aplysia californica. Biol. Cybern*. 114, 557–588. doi: 10.1007/s00422-020-00851-9 PMC8543386 33301053

[B67] Ye H. Morton D. W. Chiel H. J. (2006a). Neuromechanics of coordination during swallowing in *Aplysia californica. J. Neurosci*. 26, 1470–1485. doi: 10.1523/JNEUROSCI.3691-05.2006 PMC6675507 16452671

[B68] Ye H. Morton D. W. Chiel H. J. (2006b). Neuromechanics of multifunctionality during rejection in *Aplysia californica. J. Neurosci*. 26, 10743–10755. doi: 10.1523/JNEUROSCI.3143-06.2006 PMC6674742 17050713

[B69] Yu Z. Thomas P. J. (2021). Dynamical consequences of sensory feedback in a half-center oscillator coupled to a simple motor system. Biol. Cybern. 115, 135–160. doi: 10.1007/s00422-021-00864-y 33656573 PMC8510507

[B70] Yu Z. Thomas P. J. (2024). Variational analysis of sensory feedback mechanisms in powerstroke-recovery systems. Biol. Cybern. 118, 277–309. doi: 10.1007/s00422-024-00996-x 39249120 PMC11588830

[B71] Ziv I. Baxter D. A. Byrne J. H. (1994). Simulator for neural networks and action potentials: description and application. J. Neurophysiol. 71, 294–308. doi: 10.1152/jn.1994.71.1.294 7512628

