# Supplementary material for: NEURONpyxl: fast, flexible, Python-integrated simulation of biophysical neural networks with complex plastic synapses
Source: Front Comput Neurosci. 2026 May 19;20:1771884. doi: 10.3389/fncom.2026.1771884 (PMC13226625; doi:10.3389/fncom.2026.1771884)
Supplement: Supplementary file 1 [file Data_Sheet_1.pdf]

Supplementary Material

NEURONPYXL SPREADSHEET TUTORIAL

Biophysical Properties of Cells

Each cell in NEURONpyxl is treated as a point cell, and all units are converted from NEURON's distributed units to absolute units to match the values reported in SNNAP. Therefore, each cell is composed of only one compartment and the axial resistance, length and diameter of each cell are insignificant quantities and are hence left at their default values (Carnevale and Hines, 2006). There is also no temperature dependence. Future work could implement multi-compartment models for NEURONpyxl cells.

To add a cell, write its name in the left-most column of the region shown in Figure S1. You must also enter the membrane capacitance in μF. All of the other sections of the spreadsheet will update when cells are added to the network.

Ion Channels

In the "Neu" sheet, you can add neurons and edit parameters for the ion channels, shown also in Figure S1. The ion channels determine which ions within NEURON are used. In NEURON, you can use either Ca^{2+}, Na^{+}, Cl^{-}, K^{+} or a nonspecific current. To use either of the four ions, the name of your ion channel must start with one of the four ions (e.g. Ka, Napp, Cl, CaN). If the name of the ion channel does not start with an ion, then NEURON will use a nonspecific current. Ion channel names must start with a letter.

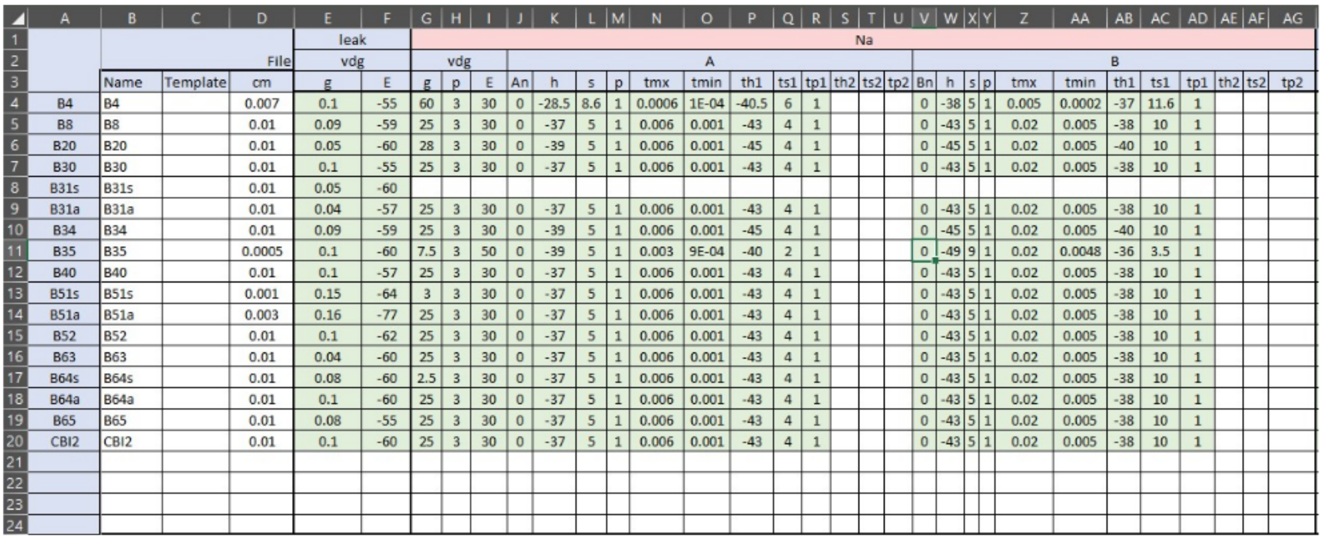

	A	B	C	D	E	F	G	H	I	J	K	L	M	N	O	P	Q	R	S	T	U	V	W	X	Y	Z	AA	AB	AC	AD	AE	AF	AG
1					leak		Na																										
2				File	vdg		vdg			A												B											
3		Name	Template	cm	g	E	g	p	E	An	h	s	p	tmx	tmin	th1	ts1	tp1	th2	ts2	tp2	Bn	h	s	p	tmx	tmin	th1	ts1	tp1	th2	ts2	tp2
4	B4	B4		0.007	0.1	-55	60	3	30	0	-28.5	8.6	1	0.0006	1E-04	-40.5	6	1				0	-38	5	1	0.005	0.0002	-37	11.6	1			
5	B8	B8		0.01	0.09	-59	25	3	30	0	-37	5	1	0.006	0.001	-43	4	1				0	-43	5	1	0.02	0.005	-38	10	1			
6	B20	B20		0.01	0.05	-60	28	3	30	0	-39	5	1	0.006	0.001	-45	4	1				0	-45	5	1	0.02	0.005	-40	10	1			
7	B30	B30		0.01	0.1	-55	25	3	30	0	-37	5	1	0.006	0.001	-43	4	1				0	-43	5	1	0.02	0.005	-38	10	1			
8	B31s	B31s		0.01	0.05	-60																											
9	B31a	B31a		0.01	0.04	-57	25	3	30	0	-37	5	1	0.006	0.001	-43	4	1				0	-43	5	1	0.02	0.005	-38	10	1			
10	B34	B34		0.01	0.09	-59	25	3	30	0	-39	5	1	0.006	0.001	-45	4	1				0	-45	5	1	0.02	0.005	-40	10	1			
11	B35	B35		0.0005	0.1	-60	7.5	3	50	0	-39	5	1	0.003	9E-04	-40	2	1				0	-49	9	1	0.02	0.0048	-36	3.5	1			
12	B40	B40		0.01	0.1	-57	25	3	30	0	-37	5	1	0.006	0.001	-43	4	1				0	-43	5	1	0.02	0.005	-38	10	1			
13	B51s	B51s		0.001	0.15	-64	3	3	30	0	-37	5	1	0.006	0.001	-43	4	1				0	-43	5	1	0.02	0.005	-38	10	1			
14	B51a	B51a		0.003	0.16	-77	25	3	30	0	-37	5	1	0.006	0.001	-43	4	1				0	-43	5	1	0.02	0.005	-38	10	1			
15	B52	B52		0.01	0.1	-62	25	3	30	0	-37	5	1	0.006	0.001	-43	4	1				0	-43	5	1	0.02	0.005	-38	10	1			
16	B63	B63		0.01	0.04	-60	25	3	30	0	-37	5	1	0.006	0.001	-43	4	1				0	-43	5	1	0.02	0.005	-38	10	1			
17	B64s	B64s		0.01	0.08	-60	2.5	3	30	0	-37	5	1	0.006	0.001	-43	4	1				0	-43	5	1	0.02	0.005	-38	10	1			
18	B64a	B64a		0.01	0.1	-60	25	3	30	0	-37	5	1	0.006	0.001	-43	4	1				0	-43	5	1	0.02	0.005	-38	10	1			
19	B65	B65		0.01	0.08	-55	25	3	30	0	-37	5	1	0.006	0.001	-43	4	1				0	-43	5	1	0.02	0.005	-38	10	1			
20	CBI2	CBI2		0.01	0.1	-60	25	3	30	0	-37	5	1	0.006	0.001	-43	4	1				0	-43	5	1	0.02	0.005	-38	10	1			
21																																	
22																																	
23																																	
24																																	

Figure S1. Table in the "Neu" sheet where cells are added to the network, membrane capacitance is defined, and ion channels and their parameters are defined.

Conductance to ion

Name	ch	ion	ch	ion	ch	ion	ch	ion
B4	CaN	Ca						
B8								
B20	Na	Na						
B30	Na	Na						
B31s								
B31a	Na	Na						
B34	Na	Na						
B35								
B40								
B51s								
B51a								
B52								
B63	Na	Na						
B64s								
B64a								
B65	Na	Na						
CBI2	Na	Na						

Ion pools

Name	ion	K1	K2	ion	K1	K2	ion	K1	K2
B4	Ca	10	9						
B8									
B20	Na	1	0.2						
B30	Na	1	0.2						
B31s									
B31a	Na	14	0.4						
B34	Na	1	0.2						
B35									
B40									
B51s									
B51a									
B52									
B63	Na	14	0.4						
B64s									
B64a									
B65	Na	1	0.2						
CBI2	Na	1	0.2						

Ion to conductance

Name	ion	ch	opt1	opt2	p1	p2	b	ion	ch	opt1	opt2	p1	p2	b
B4	Ca	CaN	3	2	60		17	Ca	Kcaf	1	1	0.01		

Figure S2. Tables in the "Neu" sheet where ion pools and ion pool regulation are defined.

A leak channel is also included by default. If you do not want to use it, simply set the conductance to 0. Lastly, leave the "Template" column empty.

Each ion channel has a name, and parameters for an A and a B component, corresponding to equations 15, 16, 17, and 18. "vdg" stands for voltage-dependent conductance. Under "vdg", you must enter values for g (conductance), p (power), and E (reversal potential), corresponding to equation 14. For A (activation) and B (inactivation) you must fill in at least An/Bn (initial value), h, s, p, and tmx. If you only enter these values, then equation 20 will be used. If you also enter values for "th1", "ts1", and "tp1", then equation 15 (A) or 17 (B) will be used. If you additionally fill in "th2", "ts2", and "tp2", then equation 16 (A) or 18 (B) will be used.

Ion Pools

Ion pools can also be added in the "Neu" sheet using the section shown in the "Ion pools" table in Figure S2. Enter the type of ion of the ion pool in the "ion" column. The ion pool will update the intracellular concentration in NEURON. The columns "K1" and "K2" correspond to the parameters in equation 22.

The ion pools must be supplied by at least one ion channel. This is defined in the "Conductance to ion" table shown in Figure S2. There can be multiple ions that feed a single pool (given by the "ch" column), and an ion pool ("ion" column) requires at least one ion channel to supply it.

To regulate an ion channel with an ion pool, enter parameters in the "Ion to conductance" table shown in Figure S2. The "ion" column is which ion pool will regulate the channel in the "ch" column. The number entered into the "opt1" column defines which of the functions in equation 23 to use. The number entered into the "opt2" column defines which of the functions in equation 24 to use. Provide values of "p1", "p2",

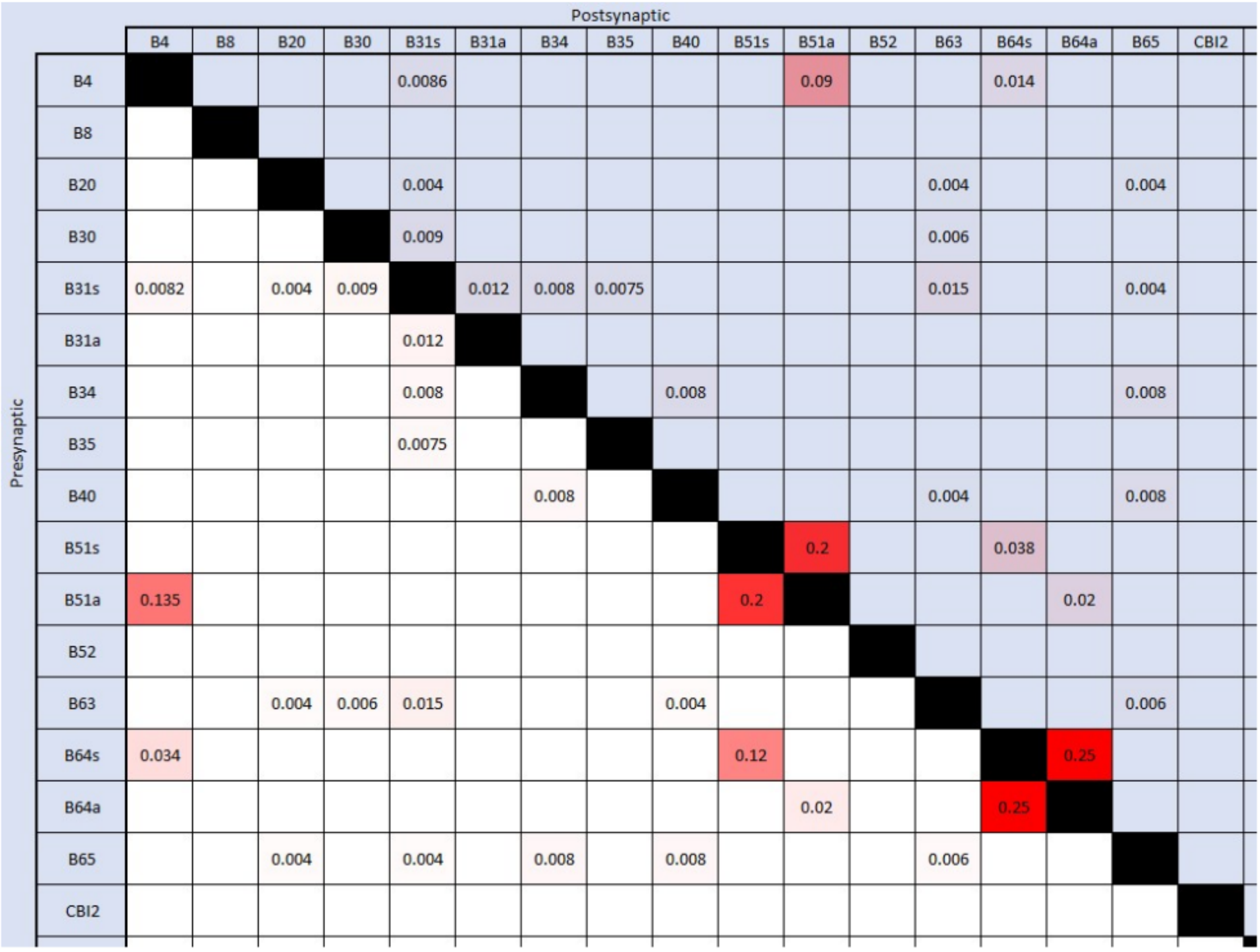

Postsynaptic

Presynaptic	B4	B8	B20	B30	B31s	B31a	B34	B35	B40	B51s	B51a	B52	B63	B64s	B64a	B65	CBI2
B4					0.0086						0.09			0.014			
B8																	
B20					0.004								0.004			0.004	
B30					0.009								0.006				
B31s	0.0082		0.004	0.009		0.012	0.008	0.0075					0.015			0.004	
B31a					0.012												
B34					0.008				0.008							0.008	
B35					0.0075												
B40							0.008						0.004			0.008	
B51s											0.2			0.038			
B51a	0.135									0.2					0.02		
B52																	
B63			0.004	0.006	0.015				0.004							0.006	
B64s	0.034									0.12					0.25		
B64a											0.02			0.25			
B65			0.004		0.004		0.008		0.008				0.006				
CBI2																	

Figure S3. Table in the “es” sheet where gap functions are defined.

and “b” as required by the equations you have selected and their corresponding units (see the section Implementing SNNAP in NEURON).

Electrical Synapses

Electrical synapses are defined in the matrix shown in Figure S3. The conductances that correspond with equation 26 are entered into the matrix. It is not required to enter a conductance for both neurons in the electrical synapse. You should not connect a neuron to itself with an electrical synapse.

Chemical Synapses

There is a similar matrix for defining chemical synapses. However, since the equations are more complex, there are now 3 matrices to fill out in order to define a complete chemical synapse. First, you must define the synaptic conductance in the “cs_g” sheet. Then, you must define the reversal potential in the “cs_E” sheet. Then you must define the synaptic activation functions in the “cs_FAT” sheet. Both the “cs_g” and “cs_E” sheets behave exactly like the “es” sheet, except there is an option for two chemical synapses within each cell of the matrix. The upper cell is called the “fast” synapse and the lower cell is called the “slow” synapse. That said, the paramter choices are still up to the user.

Presynaptic \ Postsynaptic	B4	B8	B20	B30	B31s	B31a	B34	B35	B40	B51s	B51a	B52
B4		2			2					36		2
B8												
B20	10<br>25	5<br>6										
B30		7<br>26			8							
B31s												
B31a					12							
B34		1<br>29	4		9<br>37							
B35	15<br>16										2	17<br>18
B40		2<br>19			2							
B51s												
B51a		2										20
B52		2								20<br>4		

		taus		depression		Voltage dependence				facilitation	
		u1	u2	ud	ur	h	s	p	tx	ion	u
11	1	0.01									
11	2	0.015									
3	3	0.1									
3	4	0.2									
3	5	0.004	0.0015							Na	0.5
2	6	1				-31	3	1	0.5	Na	0.5
1	7	0.2	0.001								
1	8	0.005								Na	0.5
9	9	0.01								Na	0.5
3	10	0.02									
1	11	0.5	5			-30	15	1	0.5		
2	12	0.2				-48	4	3	0.1	Na	0.5
1	13	1.5									

Figure S4. Table in the "cs_FAT" sheet where synaptic activations are defined.

The "cs_FAT" sheet is more complex. In each cell of the matrix, enter the number that corresponds to the set of equations in the table on the right side of the sheet.

In the "taus" table, the parameters "u1" and "u2" correspond to τ_1 and τ_2 in equation 29. If only "u1" is provided, then $\tau_1 = \tau_2$. This is the only activation where parameters are required.

In the "depression" table, the parameters "ud" and "ur" correspond to τ_d and τ_r in equation 31. If either "ud" or "ur" are not provided, there will be no modulation.

In the "voltage dependence" table, the parameters correspond to those in equation 33. If "tmx" is not provided, it will set voltage-dependent activation to its steady state (equation 34). If any of the other parameters are not provided, there will be no voltage-dependence.

In the "faciliation" table, the parameters correspond to those in equation 25. The synaptic activation will be modulated by an ion, with a time constant of "u". If either of the parameters are not provided, there will be no modulation.

Simulation Control

To initialize the voltages, fill in the "Initial voltage" table in the "Neu" sheet in Figure S5. Each cell should have an initialized voltage.

Initial voltage	
Name	mV
B4	-60
B8	-60
B20	-60
B30	-60
B31s	-60
B31a	-60
B34	-60
B35	-60
B40	-60
B51s	-60
B51a	-60
B52	-60

Current injection			
neuron	start	stop	magnitude
B4	0	30	-2
B8	0	30	-2
B20	0	30	-2
B30	0	30	-2
B31s	0	30	-2
B34	0	30	-2
B35	0	30	-2
B40	0	30	-2
B51s	0	30	-2

Figure S5. Tables where voltages are initialized and current clamps are defined.

To define an external stimulation, you can apply a current clamp in a sheet which has the extension ".smu". You can have any number of current clamps, as long as the neuron, amplitude of current, the start time and the end time of the clamp are provided. Any other tables in the ".smu" sheet are for SNNAP simulations, so further simulation control must be done in the command line or the Python API. For more simulation control options, see the README.md file in the GitHub repository.
