# Supplementary material for: NEURONpyxl: fast, flexible, Python-integrated simulation of biophysical neural networks with complex plastic synapses
Source: Front Comput Neurosci. 2026 May 19;20:1771884. doi: 10.3389/fncom.2026.1771884 (PMC13226625; doi:10.3389/fncom.2026.1771884)
Supplement: Supplementary file 2 [file Data_Sheet_2.pdf]

Appendix

This file contains a supplementary appendix for Dickman et al's 2026 Frontiers in Computational Neuroscience article, "NEURONpyxl: fast, flexible, Python-integrated simulation of biophysical neural networks with complex plastic synapses." Here we provide a complete list of the equations used in constructing NEURONpyxl models. The units and descriptions for the symbols in this section are provided in Table 3 in the main article.

The current-balance equation that determines the rate of change of the membrane potential is given by

$$C_{\mathrm{m}}\frac{dV_i}{dt} = I_i^{(\mathrm{app})} - \sum_j I_{ij}^{(\mathrm{vd})} - \sum_k I_{ik}^{(\mathrm{es})} - \sum_l I_{il}^{(\mathrm{cs})} + I_i^{(\mathrm{noise})} \tag{A1}$$

where C_{m} is the membrane capacitance, $I_i^{(\mathrm{app})}$ is the injected current, $I_{ij}^{(\mathrm{vd})}$ is the current from voltage-dependent ion channels in cell i for channel j, $I_{ik}^{(\mathrm{es})}$ is the current from electrical synapses in cell i based on the potential of cell k, and $I_{il}^{(\mathrm{cs})}$ is the current from chemical synapses in cell i from cell l. $I_i^{(\mathrm{noise})}$ is the sum of two independently fluctuating currents (one excitatory and the other inhibitory) with the timing defined by a Poisson process (see Noise Implementation). The neurons are Hodgkin-Huxley type and the voltage-dependent ion channel currents are given by

$$I_{ij}^{(\mathrm{vd})} = \bar{g}_{ij} A^p(t, V_i) B^q(t, V_i)(V_i - E_{ij}) f[\mathrm{reg}] \tag{A2}$$

Here, i refers to the cell and j refers to the ion channel, $\bar{g}_{ij}$ is the maximal conductance of the synapse, q is either 0 or 1, and $p \geq q$. The quantity A is the activation gating variable and B is the inactivation gating variable. V_i is the membrane potential and E_{ij} is the reversal potential of the channel. The final component of the equation is $f[\mathrm{reg}]$, which is the regulator function that depends on the concentration of an ion pool. In SNNAP, ion regulation can be a product of multiple regulator functions (Ziv et al., 1994), but for simplicity, NEURONpyxl only allows for regulation by one ion per channel.

Activation Functions

$A(t, V)$, the ion channel activation gating variable, obeys the differential equation

$$\frac{dA(t, V)}{dt} = \frac{A_\infty(V) - A}{\tau(V)}, \qquad \text{with} \tag{A3}$$

$$A_\infty(V) = \frac{1 - A_{\mathrm{min}}}{\left(1 + \exp\left(\frac{h-v}{s}\right)\right)^r} + A_{\mathrm{min}}, \tag{A4}$$

where A_{min} is the minimum activation, h is the half-activation voltage, and s is the slope factor (see main text, Tab. 3), and r is a shaping parameter of the Boltzmann equation. $B(t, V)$, the ion channel inactivation

gating variable, obeys the differential equation

$$\frac{dB(t,V)}{dt} = \frac{B_\infty(V) - B}{\tau(V)}, \qquad \text{with} \tag{A5}$$

$$B_\infty(V) = \frac{1 - B_{\text{min}}}{\left(1 + \exp\left(\frac{V-h}{s}\right)\right)^r} + B_{\text{min}}. \tag{A6}$$

The general form of the time constants is given by

$$\tau(V) = \tau_{\text{min}} + \frac{\tau_{\text{max}} - \tau_{\text{min}}}{\left(1 + \exp\left(\frac{V-h_1}{s_1}\right)\right)^{r_1} \left(1 + \exp\left(\frac{V-h_2}{s_2}\right)\right)^{r_2}} \tag{A7}$$

In some cases, $r_2 = 0$ and $r_1 > 0$, in which case Eq. (A7) reduces to a single sigmoidal function. If any of h_n, s_n, and r_n are not provided for τ, then $\tau = \tau_{\text{max}}$. If τ_{max} is not provided, then we replace A and B as functions of time with their asymptotic limits (i.e. the singular limit, taking $\tau_{\text{max}} \to 0$), namely

$$A \leftarrow A_\infty(V) \tag{A8}$$

$$B \leftarrow B_\infty(V) \tag{A9}$$

Ion Pool Regulation

SNNAP allows for several mechanisms of ion pool regulation. Our NEURONpyxl implementation includes the following cases. Other ion pool regulation mechanisms may be incorporated as needed, thanks to the modularity and customizability built into NEURON.

In NEURON, the available ions are Ca^{2+}, Na^{+}, K^{+}, and Cl^{-}. The ion pools are fed by ion channels. The concentration of a given ion pool is determined by the total current from all of the ion channels feeding it

$$\frac{d[\text{ion}]_i}{dt} = k_1 \left[-k_2 \sum_j I_{ij}^{(vd)} - [\text{ion}]_i \right] \tag{A10}$$

SNNAP includes provisions for modulation of conductances by intracellular ion pools and second messengers (Ziv et al. (1994), Eqs. A1 and A2). In NEURONpyxl, a factor "reg" can regulate conductances through any of the following formulae:

$$f[\text{reg}] = \begin{cases} \text{reg} \\ 1 + \text{reg} \\ \frac{1}{1+\alpha\cdot\text{reg}} \\ 1 & \text{(No regulation)} \end{cases} \tag{A11}$$

where α is a user-specified constant. There are several options for reg. The explicitly defined options are

$$\text{reg}([\text{ion}]) = \begin{cases} \frac{[\text{ion}]}{\beta+[\text{ion}]} \\ \\ 1 + \frac{[\text{ion}]}{\beta+[\text{ion}]} \\ \\ \frac{1}{1+\beta\cdot[\text{ion}]} \\ \\ \exp\left(\frac{\beta+[\text{ion}]}{\tau_{\text{reg}}}\right) \end{cases} \tag{A12}$$

where β and τ_{reg} are scaling parameters specified in units such that reg is unitless. There is also an option to define reg implicitly in the form of an ODE of which the solution is a decaying exponential:

$$\frac{d\,\text{reg}}{dt} = \frac{\gamma[\text{ion}] - \text{reg}}{\tau_{\text{reg}}} \tag{A13}$$

with default initial condition $\text{reg}(0) = 0$; τ_{reg} is a time constant, and $\gamma = 10^6\ \text{mM}^{-1}$ is a scale factor.

Chemical Synapses and Secondary Messengers

We model an electrical synapses as a gap junction, such that the current from cell j into cell i is given by

$$I_{ij}^{(\text{es})} = \bar{g}_{ij}(V_i - V_j) \tag{A14}$$

where $\bar{g}_{ij}$ is the conductance from cell j to cell i.

For chemical synapses the synaptic current of the postsynaptic cell i due to the activation of the presynaptic neuron j is given by

$$I_{ij}^{(\text{cs})} = \bar{g}_{ij}A^{(\text{cs})}(t, V_i)(V_i - E_{ij}) \tag{A15}$$

The activation, $A^{(\text{cs})}$, is the product of a time-dependent (and voltage-independent) activation factor $M_1(t)$ and a time- and voltage-dependent activation factor $M_2(t, V)$. The time-dependent activation includes a modulatory term $X(t, [\text{ion}])$, which is the product of modulation by an ion pool $f[\text{reg}]$ and by presynaptic spiking (PSM). NEURONpyxl supports modeling synaptic facilitation and depression by $f[\text{reg}]$ and only synaptic depression by PSM. For synapses without plasticity, $X = 1$. The equations that determine these

synaptic activations depend on whether the presynaptic cell initiates a spike.

$$A^{(\mathrm{cs})}(t,V) = M_1(t)\cdot M_2(t,V) \tag{A16}$$

$$\frac{d^2M_1(t)}{dt^2} = \frac{X-(\tau_1+\tau_2)dM_1/dt - M_1}{\tau_1\tau_2} \tag{A17}$$

$$X(t,[\mathrm{ion}]) = \begin{cases} \mathrm{PSM}(t)\cdot f[\mathrm{reg}(t,[\mathrm{ion}])] & \text{presynaptic cell spiking} \\ 0 & \text{presynaptic cell is not spiking} \end{cases} \tag{A18}$$

$$\frac{d\mathrm{PSM}(t)}{dt} = \begin{cases} -\frac{\mathrm{PSM}}{\tau_\mathrm{d}} & \text{depression, if presynaptic cell spiking} \\ \frac{1-\mathrm{PSM}}{\tau_\mathrm{r}} & \text{recovery, if presynaptic cell not spiking} \\ 0 & \text{no modulation} \end{cases} \tag{A19}$$

$$f[\mathrm{reg}] = \begin{cases} 1+\mathrm{reg} & \text{synaptic facilitation} \\ 1 & \text{no facilitation} \end{cases} \tag{A20}$$

The PSM factor, which ranges from 0 to 1, follows kinetics governed by depression time constant τ_d when the presynaptic cell is spiking, and by recovery time constant τ_r when the presynaptic cell is not spiking. If no modulation is included in the model, then PSM is set to one (constant). If modulation is included, then the default initial condition for PSM is $\mathrm{PSM}(0) = 1$. The time-dependent activation is modeled as a second-order differential equation with time constants τ_1 and τ_2. If there is facilitation term $f[\mathrm{reg}]$, then reg is given by Eq. A13, and $[\mathrm{ion}]$ is the concentration of the ion in the presynaptic neuron.

If a synapse is voltage-dependent, then the voltage-dependent activation of a synapse is $M_2(t,V_i)$, where $M_2(t,V)$ is given by one of the following:

$$\begin{cases} M_2(t,V) = M_{2,\infty}(V) \\ \frac{dM_2(t,V)}{dt} = \frac{M_{2,\infty}(V)-M_2}{\tau_\mathrm{v}} \end{cases} \tag{A21}$$

where

$$M_{2,\infty}(V) = \frac{1}{(1+\exp((h_\mathrm{v}-v)/s_\mathrm{v}))^{r_\mathrm{v}}}. \tag{A22}$$
